# Identification of quantitative trait loci controlling nitrogen use efficiency-related traits in rice at the seedling stage under salt condition by genome-wide association study

**DOI:** 10.3389/fpls.2023.1197271

**Published:** 2023-07-27

**Authors:** Nhung Thi Hong Phan, Xavier Draye, Cuong Van Pham, Pierre Bertin

**Affiliations:** ^1^ Earth and Life Institute, Université Catholique de Louvain, Louvain-la-Neuve, Belgium; ^2^ Agronomy Faculty, Vietnam National University of Agriculture, Hanoi, Vietnam

**Keywords:** GWAS, 3K Rice Genomes Project, NUE, saline, dry weight

## Abstract

Rice cultivation is facing both salt intrusion and overuse of nitrogen fertilizers. Hence, breeding new varieties aiming to improve nitrogen use efficiency (NUE), especially under salt conditions, is indispensable. We selected 2,391 rice accessions from the 3K Rice Genomes Project to evaluate the dry weight under two N concentrations [2.86 mM – standard N (SN), and 0.36 mM – low N (LN)] crossed with two NaCl concentrations [0 (0Na) and 60 mM (60Na)] at the seedling stage. Genome-wide association studies for shoot, root, and plant dry weight (DW) were carried out. A total of 55 QTLs – 32, 16, and 7 in the whole, *indica*, and *japonica* panel – associated with one of the tested traits were identified. Among these, 27 QTLs co-localized with previously identified QTLs for DW-related traits while the other 28 were newly detected; 24, 8, 11, and 4 QTLs were detected in SN-0Na, LN-0Na, SN-60Na, and LN-60Na, respectively, and the remaining 8 QTLs were for the relative plant DW between treatments. Three of the 11 QTLs in SN-60Na were close to the regions containing three QTLs detected in SN-0Na. Eleven candidate genes for eight important QTLs were identified. Only one of them was detected in both SN-0Na and SN-60Na, while 5, 0, 3, and 2 candidate genes were identified only once under SN-0Na, LN-0Na, SN-60Na, and LN-60Na, respectively. The identified QTLs and genes provide useful materials and genetic information for future functional characterization and genetic improvement of NUE in rice, especially under salt conditions.

## 1 Introduction

Rice is a staple food for more than half of the world’s population. Approximately 75% of the total area of harvested rice in the world is cultivated in South and South-East Asia countries (FAO, 2021). However, these regions are facing two severe environmental concerns: salt intrusion and overuse of nitrogen fertilizers (Heffer and Prud’homme, 2017; Smajgl et al., 2015; Eckstein et al., 2019; Wassmann et al., 2019). In the past five decades, N fertilizer consumption is an approximately 5-fold increase in the world, with a 2-fold and 15-fold increase in developed and developing countries, respectively (IFA, 2019). Among them, approximately 20% of the total N fertilizers are used for rice (Ladha et al., 2005). However, rice plants could absorb less than half of the N applied, and the rest is wasted in soil, water, and the atmosphere (Lea and Miflin, 2003; Ladha et al., 2005; Udvardi et al., 2021). Therefore, improving nitrogen use efficiency (NUE) for rice under saline conditions has become of primary importance.

NUE is defined as the yield (grain, starch, biomass, depending on the authors) per N applied unit. It can be partitioned into two processes within the plant: absorption NUE (that refers to the capacity of the plant to absorb the applied N) and physiological NUE (that refers to its efficiency to use the absorbed N for metabolic purposes) (Ladha et al., 2005; Murtaza et al., 2013; Nguyen et al., 2014; Beatty and Good, 2018). NUE is strongly correlated with plant growth and development affecting root morphology, tiller number, biomass, and yield, and is governed by multiple interacting genetic and environmental factors (Ladha et al., 2005; Nguyen et al., 2016; Sharma et al., 2021; Sandhu et al., 2021; Phan et al., 2023). Therefore, research on NUE has often been related to these traits, especially dry weights. Identification of the QTLs (quantitative trait loci) associated with NUE, or NUE-related traits is an important step to improve NUE in rice.

Salinity reduces NUE by affecting all processes of N metabolism in the plant, thus causing a severe decline in crop production. Salt inhibits NO_3_^-^, N content, glutamine synthetase, and nitrate reductase but enhances NH_4_^+^ content and glutamate synthase in rice (Hoai et al., 2003; Wang et al., 2012; Phan et al., 2023). Notably, NH_4_^+^ increase does not always bring advantages because high NH_4_^+^ uptake cannot always be assimilated, inducing ion toxicity and decrease in NUE (Ouyang et al., 2010). The subsequent decrease in N uptake and assimilation causes a decrease in photosynthesis, antioxidant enzymes activity, dry weight, and grain yield (Abdelgadir et al., 2005; Wang et al., 2012; Chen et al., 2022; Phan et al., 2023). The effects of salt on rice may alter the expression of some genes controlling N uptake and N assimilation: *OsNRT* family, *OsAMT* family, *OsNR1*, *OsGS1.2*, *OsNADH-GOGAT*, or *OsFd-GOGAT* (Wang et al., 2012; Rohilla and Yadav, 2019; Huang et al., 2020; Decui et al., 2022). Interestingly, some genes controlling N uptake were found to be associated with salt tolerance/susceptibility in rice. Shi et al. (2017) detected that *OsNRT2.2*, associated with nitrogen uptake in rice, was also related to salt susceptibility, whereas Batayeva et al. (2018) elucidated that *OsAMT1.3*, regulating ammonium transport, was also associated with salt tolerance under severe salinity stress. In previous studies, we demonstrated that applying high N rates under saline condition in rice did not result in a significant increase in grain yield but caused significant reduction in both NUE and dry weight; NUE was reduced by increasing either salinity or nitrogen levels, and the reduction was mainly linked to absorption NUE (Phan et al., 2017, 2023).

Previously, a large number of QTLs for NUE and its related traits have been detected under different N concentrations by using bi-parental linkage mapping populations (Fang and Wu, 2001; Lafitte et al., 2002; Price et al., 2002; Hittalmani et al., 2003; Lian et al., 2005; Li et al., 2005; Nguyen et al., 2016; Zhou et al., 2017). Indeed, eight QTLs for plant height were identified under low or high N levels (5 and 40 mg L^-1^) in a double-haploid population consisting of 123 lines derived from a cross between IR64 and Azucena (Fang and Wu, 2001). Genomic regions for plant height, dry weight, and relative dry weight under two N treatments (1N - normal N and 1/6N - low N level) were identified by using 239 RILs from a cross between Zhenshan 97 and Minghui 63 (Lian et al., 2005). Also, 14 QTLs for NUE component traits and 63 QTLs for NUE-related traits were identified under three N levels (1N, 1/4N, and 1/8N) in hydroponics by using 174 RILs from the cross IR64/Azucena (Nguyen et al., 2016). Such bi-parental mapping method presents high statistical power due to using many individuals sharing an identical genotype at a given locus; however, it has a low resolution because of the limited number of recombination events in the development of the population.

In recent years, a lot of QTLs or candidate genes have been identified by genome-wide association study (GWAS) by using high-density genome-wide single nucleotide polymorphism (SNP) detected by next-generation sequencing of unrelated individuals in a population. The detected QTLs showed a high resolution due to the long recombination histories of natural populations (Shi et al., 2017; Batayeva et al., 2018; Naveed et al., 2018; Liu et al., 2019). A combination of GWAS, gene annotation based on high-quality reference genomes, and haplotype analysis is an effective way to identify candidate genes for the tested traits and elite materials that are useful for future breeding and molecular dissection for rice (Wang et al., 2017; Naveed et al., 2018; Norton et al., 2021). Regarding NUE traits, *OsNAC42, OsNPF6.1*, and *OsNLP4* have been detected by GWAS in rice recently (Tang et al., 2019; Yu et al., 2021). GWAS can be applied in a large amount of genotypes and save time compared to conventional methods. In the last decade, 29 million SNPs were discovered by sequencing 3,010 rice accessions from 89 countries in the 3,000 Rice Genomes Project (3K RGP), providing useful information for genetic research and breeding (3K RGP, 2014; Li et al., 2014). However, most QTLs and genes mentioned above have been detected under non-saline conditions. To date, no GWAS has been conducted yet to examine a large rice population for NUE-related traits under saline condition. Using the 3K RGP database, GWAS was used to detect a large number of QTLs related to agronomical traits: heading date, seedling length, 100-grain weight, grain width, grain length, culm diameter, culm length, culm number, leaf width, leaf length, leaf angle, panicle type, [https://snp-seek.irri.org/_gwas.zul]. We focused on finding genetic information related to the dry weight which is one of the most important NUE-related traits, under varying N and NaCl treatments.

## 2 Materials and methods

### 2.1 Plant growth

We selected 2,391 rice accessions from 75 countries from the 3,000 RGP (**Supplementary Table S1**; 3K RGP, 2014; Li et al., 2014) for the main experiment (main EXP). Then, a selection of 1,332 accessions from 68 countries was realized based on low relatedness criteria revealed by the phylogenetic tree, in order to realize a confirmatory experiment (confirmatory EXP). It was created by Archaeopteryx Tree image in TASSEL 5.2.57 by using the neighbor-joining cladogram function of 2,391 accessions with 5,902 SNPs (Bradbury et al., 2007) (**Supplementary Figure S1**). According to the 3K RGP, the accessions belonged to nine variety types, *viz. aromatic*, *aus*, *temperate japonica*, *tropical japonica*, *subtropical japonica*, *indica-*1A, *indica-*1B, *indica-*2, and *indica-*3, and the admixture (*japonica-*admixed, *indica-*admixed, and admixed). Confirmatory EXP was conducted with fewer accessions than the main EXP aiming to reduce the competition between the genotypes by increasing the space between the plants.

### 2.2 Main experiment

A hydroponic experiment was performed with 2,391 rice accessions in a phytotron at the Université Catholique de Louvain, Belgium, from March to April 2019. The seeds were sown directly in holes on extruded polystyrene plates floating in 40L tanks, 74.5cm length x 54.5 cm width x 10.0 cm height, at a density of eight seeds of the same genotype per hole, 630 holes per tank with each hole for each genotype. The tanks contained the Yoshida solution (Yoshida et al., 1976) which was renewed at 7, 10, 14, and 17 days after the treatment. The pH was adjusted daily between 5.0 and 5.5 with KOH 2M or HCl 1M. One week after sowing, five uniform plants per hole were maintained up to the end of the experiment. The climatic conditions in the phytotron were maintained at 30°C/25°C day/night, 85%-95% relative humidity, 12h photoperiod, and 210 µmol m^-2^ s^-1^ photon flux density at the top of the tanks.

At the sowing time, rice seeds were put in the Yoshida solution with two different N concentrations: standard N (SN, 2.86 mM N) and low N (LN, 0.38 mM N). At the same time, two salinity levels, viz. 0 mM NaCl (0Na) and 60 mM NaCl (60Na) were applied in the solutions. Thus, two N concentrations crossed with two NaCl levels led to a total of four treatments. Each treatment was carried out with 2,391 accessions; thus 4 tanks were used for each treatment. The experimental design was laid out as an augmented RCBD (Federer and Raghavarao, 1975) with four replications, comprising each of 4 tanks per treatment. Among the 2,391 accessions, 2,379 appeared only once (*i.e.* 8 seeds at sowing but remaining 5 plants in one hole) in each replicate, thus 594-595 accessions per tank. The 12 remaining accessions were replicated once in each of the four tanks. The 23-24 remaining holes in each tank were filled by three unsequenced genotypes to estimate the effect of the tanks. Hence, 4 treatments were conducted in 16 plates/tanks, and 50,400 plants. The tanks were moved in the phytotron twice a week. The plants were harvested after 21 days of treatment.

### 2.3 Confirmatory experiment

The confirmatory EXP was conducted to confirm the results of the main experiment, in an experimental design allowing lower the competition between plants. It was carried out in a greenhouse at UCLouvain, Belgium, from November to December 2019. The two N concentrations and NaCl levels were maintained as in the main EXP, whereas the number of accessions, plant density, duration of treatment, and climatic conditions were modified.

To reduce the competition between the accessions, we increased the space between the plants; thus, the number of accessions was reduced to 1,332 instead of 2,391 accessions in the main EXP, because of space constraints. Three seeds of each accession were sown in each hole of the plate, then two uniform seedlings were maintained after 5 days. Each plate – each tank – contained 360 holes with each genotype per hole. The space between the holes was 3 cm x 3.3 cm. Four tanks were used for each treatment. Among the 1,332 accessions from 3K RGP, 1,316 appeared only once, thus 329 accessions per tank. The 16 remaining accessions were replicated once in each of the four tanks. The other 15 remaining holes in each tank were filled by three unsequenced genotypes to calculate the effect of the tanks. In total, the experiment was conducted in 16 tanks, and 11,520 plants and was laid out as an augmented RCBD (Federer and Raghavarao, 1975). The tanks were moved inside the greenhouse twice a week.

The climatic conditions were the same as in the main EXP, except for the photoperiod which was set to 16h, in order to speed up growth. The solution treatments were applied and renewed as in the main EXP up to 14 days, then the plants were harvested after 17 days of treatment, which was adequate for screening.

### 2.4 Phenotyping

Shoot dry weight (SDW) and root dry weight (RDW) of each accession were determined after 21 days in the main EXP and 17 days in the confirmatory EXP. The shoot and root samples were collected individually, and oven-dried (48h at 70°C), and then dry weights were determined. Then, the data of SDW and RDW of each treatment were adjusted by augmentedRCBD package in R software (Aravind et al., 2020) based on the check-varieties in both EXPs.

Plant dry weight (PDW) is the sum of SDW and RDW of the same plant.

Shoot/root ratio (SRR) was calculated by SDW per RDW of each accession.

Relative plant dry weight (RePDW) was calculated by the following formulas:


(1) $$\text{RePD}{\text{W}}_{\text{LN}-0\text{N}/\text{SN}-0\text{Na}}=\text{ PD}{\text{W}}_{\text{LN}-0\text{Na}}/\text{ PD}{\text{W}}_{\text{SN}-0\text{Na}}$$

(2) $$\text{RePD}{\text{W}}_{\text{SN}-60\text{Na}/\text{SN}-0\text{Na}}=\text{ PD}{\text{W}}_{\text{SN}-60\text{Na}}/\text{ PD}{\text{W}}_{\text{SN}-0\text{Na}}$$

(3) $$\text{RePD}{\text{W}}_{\text{LN}-60\text{Na}/\text{LN}-0\text{Na}}=\text{ PD}{\text{W}}_{\text{LN}-60\text{Na}}/\text{ PD}{\text{W}}_{\text{LN}-0\text{Na}}$$

(4) $$\text{RePD}{\text{W}}_{\text{LN}-60\text{Na}/\text{SN}-60\text{Na}}=\text{ PD}{\text{W}}_{\text{LN}-60\text{Na}}/{\text{ PDW}}_{\text{SN}-60\text{Na}}$$

### 2.5 Genome-wide association study

A total of 1,011,601 GWAS SNPs were downloaded from the Rice SNP-Seek Database [https://snp-seek.irri.org, (Mansueto et al., 2016)]. We selected the SNPs with a minor allele frequency > 5% and missing rates< 5%, resulting in 588,792 SNPs. Subsequently, we randomly selected 40% of these SNPs and got 235,210 SNPs.

The GWAS was performed with a Factored Spectrally Transformed Linear Mixed Model (FaST-LMM) by FaST-LLM software (Lippert et al., 2011). We randomly selected 2.5% of these SNPs for measuring genetic similarities between the accessions. Principal components analysis of the 588,792 SNPs was done with the default setting by PLINK 1.9 (Purcell et al., 2007). Then, the eigenvalue of the top three components was selected as covariate data. The significant threshold was set at p ≤ 0.0001 (-log_10_p-value ≥ 4).

### 2.6 Linkage disequilibrium decay

We analyzed LD decay in four populations, *viz.* whole panel 1 with 2,391 accessions, whole panel 2 with 1,332 accessions, *indica* panel 1 with 1,418 accessions, *japonica* panel 1 with 652 accessions, *aus* panel 1 with 182 accessions, and *aromatic* panel 1 with 66 accessions. Random selection of 20% of the 588,792 SNPs was used to calculate the LD decay rate. We calculated r^2^ as an estimation of LD using PLINK software version 1.9 (Purcell et al., 2007). The syntax was “–r2 –ld-window-kb 1000 –ld-window 9999 –ld-window-r2 0”. Marker pairs were grouped into bins of 1 kb and the average r^2^ value of each bin was calculated. The LD decay rate was measured as a distance at which the average r^2^ dropped to half of its maximum value (Huang et al., 2010; Shi et al., 2017).

### 2.7 Haplotype analysis and candidate gene identification

The process to identify candidate genes was described by Wang et al. (2017). Multiple significant SNPs that were in a range of linkage disequilibrium (LD) decay rates were considered as a single QTL. Among all detected QTLs, we focused on the most important QTLs to identify candidate genes by gene-based association analysis and focused on the QTLs detected in both whole panels instead of in either *indica* or *japonica* panels. The most important QTLs were selected when they met at least one of the two following criteria: either consistently identified in both EXPs or close to previously reported cloned genes or fine-mapped QTL. We identified candidate genes for each important QTL in the following steps. Firstly, we identified all genes located inside the important QTL region (± 100 kb from lead SNP) from the Rice Genome Annotation Project database [http://rice.uga.edu/cgi-bin/gbrowse/rice/ (Kawahara et al., 2013)]. Secondly, all available SNPs inside these QTLs were searched from the 32 M SNPs data generated from 3 K RGP in the Rice SNP-Seek Database [https://snp-seek.irri.org, (Mansueto et al., 2016)]. Thirdly, all SNPs with minor allele frequency less than 0.05 and missing rate over 5% were removed to maintain only high-quality SNPs which were used to analyze GWAS by the multi-locus GWAS analysis [mrMLM package in R software (Zhang, 2019)]. The threshold was defined as -log_10_p-value ≥ 3 (Naveed et al., 2018). Then, for each candidate gene, we assembled the different haplotypes based on all polymorphic SNPs contained in the gene region by using PLINK software version 1.9 (Purcell et al., 2007). Finally, we tested the significance of phenotypic differences among major haplotypes (containing more than 10 accessions per haplotype) through ANOVA with the *post-hoc* Tukey HSD test. The genes with a significance level p-value< 0.05 and significantly different between the major haplotypes in both EXPs were considered to be candidate genes of the target traits.

### 2.8 Statistical analysis

Pearson’s correlation coefficients between pairs of tested traits, ANOVA, and Tukey’s tests were conducted by using R software ver.3.4.2 (R Development Core Team, 2019).

## 3 Results

### 3.1 Phenotypic variation and traits correlations

**Figure 1:**
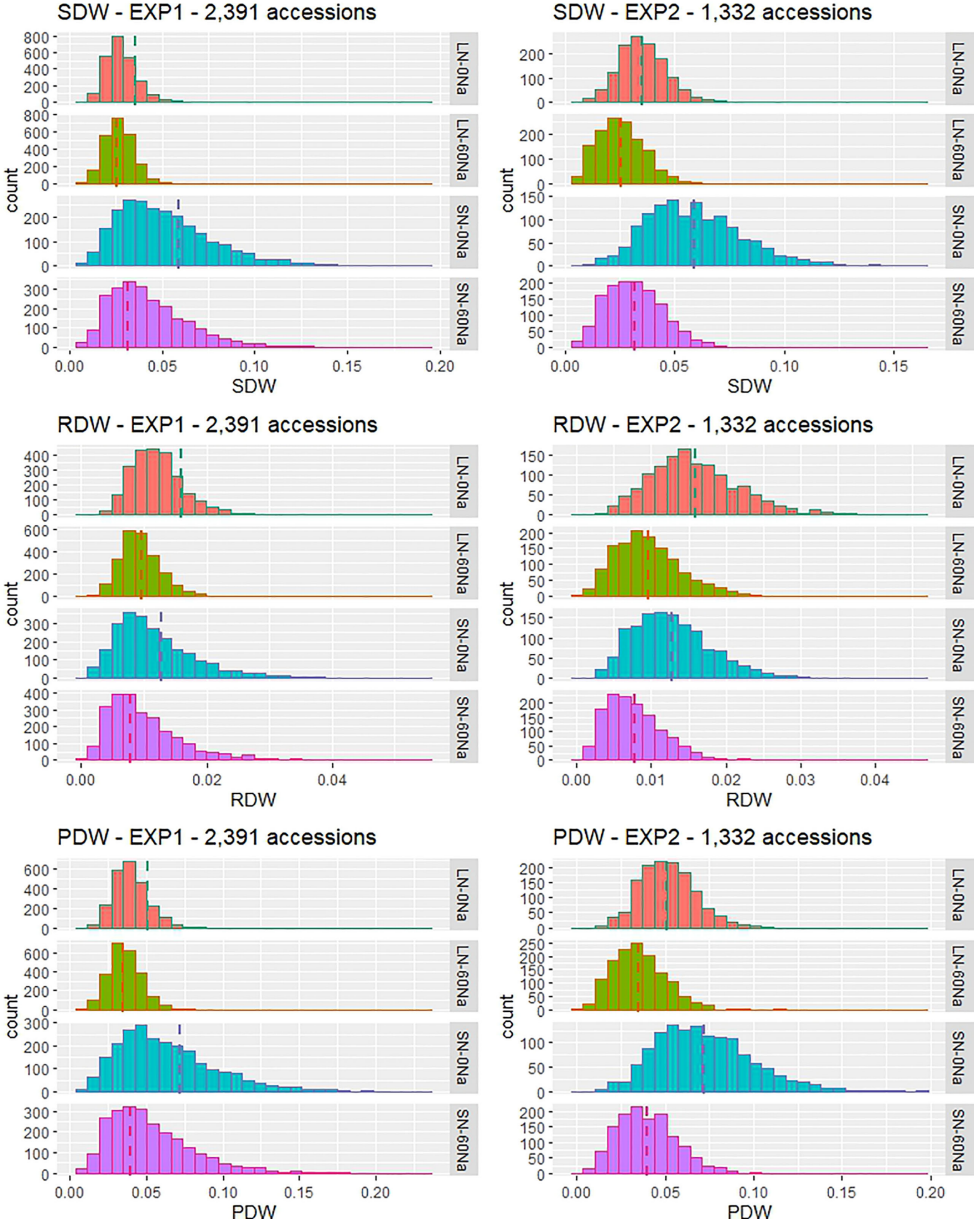
Frequency distribution of phenotypic values for SDW, RDW, and PDW in four treatments in two hydroponic experiments on the Yoshida et al. (1976) solution with 2,391 accessions (the main EXP) or 1,332 accessions (the confirmatory EXP) from the 3K Rice Genomes Project. LN: low N, 0.36 mM N, SN: standard N, 2.86 mM N, 0Na: no NaCl added, 60Na: 60 mM NaCl added, dashed line: mean value of the DWs in each treatment.

There was a wide range of variation for SDW, RDW, and PDW traits among the tested accessions, and the variation differed depending on N and NaCl treatments (**Table 1** and **Figure 1**). Among them, SN-60Na performed the largest variation in SDW, followed by SN-0Na (in the main EXP) and LN-60Na (in the confirmatory EXP), and finally the LN-0Na treatment. Among the accessions, Babaomi, Molok, Aomierte 168, Local::IRGC 53300-1, Nona Bokra, ARC 11867, Xitto, Moddai Karuppan, Dawk Put, Hansraj, and Parn A 191 produced much higher SDWs than the average value in the different treatments.

**Table 1:** SDW, RDW, and PDW of rice accessions under different N and NaCl treatments at the seedling stage in the two experiments.

Trait	Treatment	The main EXP			The confirmatory EXP		
		Mean ± SD (g)	Range (g)	CV (%)	Mean ± SD (g)	Range (g)	CV (%)
SDW	SN-0Na	0.052 ± 0.026	0.004–0.190	50.18	0.059 ± 0.022	0.011–0.160	37.63
	LN-0Na	0.028 ± 0.008	0.007–0.063	28.64	0.035 ± 0.011	0.009–0.076	30.04
	SN-60Na	0.043 ± 0.022	0.003–0.179	51.11	0.032 ± 0.013	0.004–0.097	41.78
	LN-60Na	0.027 ± 0.008	0.005–0.058	29.29	0.025 ± 0.010	0.003–0.071	41.16
RDW	SN-0Na	0.012 ± 0.007	0.002–0.056	58.11	0.013 ± 0.005	0.002–0.040	42.75
	LN-0Na	0.012 ± 0.004	0.002–0.042	35.27	0.016 ± 0.006	0.002–0.040	38.30
	SN-60Na	0.010 ± 0.006	0.002–0.047	63.08	0.008 ± 0.004	0.002–0.031	51.67
	LN-60Na	0.009 ± 0.003	0.002–0.031	35.15	0.010 ± 0.005	0.002–0.047	48.89
PDW	SN-0Na	0.063 ± 0.032	0.005–0.229	50.83	0.072 ± 0.027	0.013–0.198	37.71
	LN-0Na	0.040 ± 0.011	0.012–0.092	28.42	0.051 ± 0.016	0.014–0.107	30.69
	SN-60Na	0.053 ± 0.028	0.004–0.221	52.75	0.039 ± 0.017	0.004–0.128	42.74
	LN-60Na	0.036 ± 0.011	0.007–0.084	29.21	0.035 ± 0.015	0.003–0.118	42.00

SD, standard deviation; CV, coefficient of variation; SN-0Na, no NaCl added and standard N concentration; LN-0Na, no NaCl added and low N concentration; SN-60Na, 60 mM NaCl added and standard N concentration; LN-60Na, 60 mM NaCl added and low N concentration.

The main EXP: 2,391 accessions; the confirmatory EXP: 1332 accessions.

The correlations among the traits in different treatments of the whole panel 1 (2,391 accessions in the main EXP) and panel 2 (1,332 accessions in the confirmatory EXP) were calculated (**Supplementary Table S2C**). In both EXPs, there were strong correlations between SDW, PDW, and RDW. Among the four treatments, the correlation between SDW and RDW in LN-0Na (0.69 in the main EXP and 0.75 in the confirmatory EXP) was weaker than those in the three other treatments (0.90 and 0.87, 0.93 and 0.87, 0.76 and 0.86 in SN-0Na, SN-60Na, and LN-60Na in the main and confirmatory EXP, respectively).

N, NaCl and their interactions showed the significant effect on all the dry weights (**Supplementary Tables S2A, B**). SDW decreased with either N-deficiency or NaCl application. RDW, however, was reduced by the presence of NaCl but increased under N-deficiency treatment. Compared with the results of the main EXP, DWs in LN-0Na in the confirmatory EXP were slightly higher, and DWs in SN-60Na slightly lower than in the main EXP. DWs in LN-60Na and SN-0Na in both EXPs were similar (**Table 1**).

The four genetic subgroups - *indica*, *japonica*, *aromatic*, and *aus* – showed similar trends in both EXPs. Comparing these subgroups with each other, SDW of *indicas* was the highest, followed by *japonicas*, and then by *aus* under SN-0Na – the standard Yoshida solution – in both EXPs. SDW of the *aromatic* accessions was much lower than that of the *indica* type in the main EXP, whereas there were similar in the confirmatory EXP (**Supplementary Figure S3**). Under both LN-0Na and SN-60Na, *indica* and *aromatic* showed the highest SDW, followed by *japonica*, and finally *aus*. Under LN-60Na, the highest SDW was found in *aromatic*, followed by both *indica* and *japonica*, and finally *aus*. Thus, the *aromatic* accessions appear to better resist the simultaneous decrease in N and rise in NaCl application than the other genetic subgroups, as far as PDW is concerned, whereas *aus* accessions always showed the lowest PDWs.

### 3.2 Principal component analysis and LD decay

**Figure 2:**
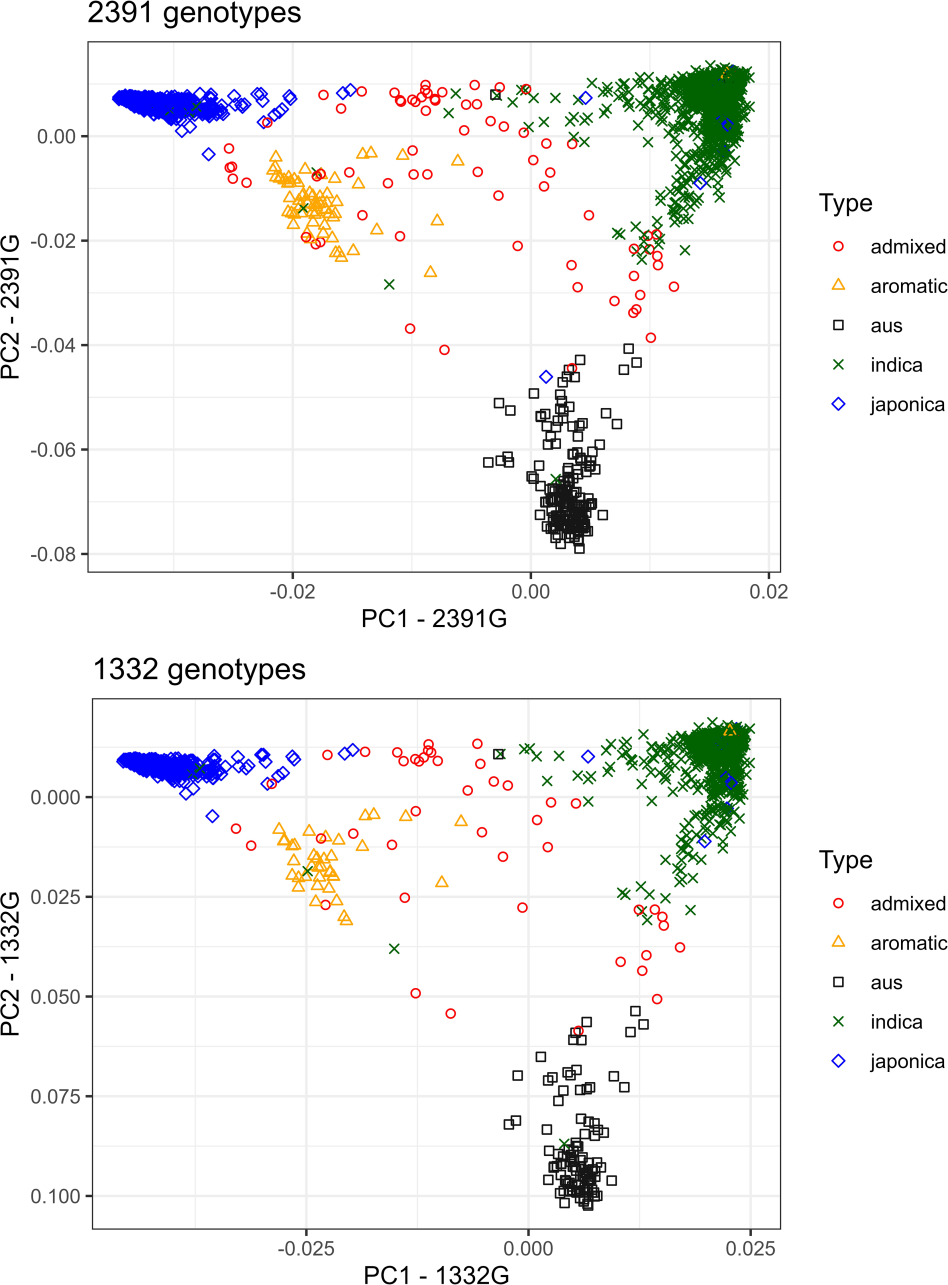
PCA plot (PC1 and PC2) of five genetic groups of rice accessions (the four *Oryza sativa* subspecies *indica, japonica*, *aus*, and *aromatic* and admixed accessions) using 588,792 SNPs from the 3K Rice Genomes Project. Left figure: PCA of 2,391 genotypes; right figure: PCA of the subset of 1,332 selected genotypes.

The principal component analysis of 2,391 and 1,332 accessions classified them into four main subgroups: *indica*, *japonica*, *aromatic*, and *aus* (**Figure 2**). The admixed accessions spread out the whole plot. The top three principal components (PC) of the analysis of 2,391 accessions explained 43.1 (PC1), 17.2 (PC2), and 7.3% (PC3) of the total variation while those of the analysis of 1,332 accessions explained 43.0, 17.3 and 7.4% of the total variation. Hence, the population structure should be considered in the following GWAS analysis.

**Figure 3:**
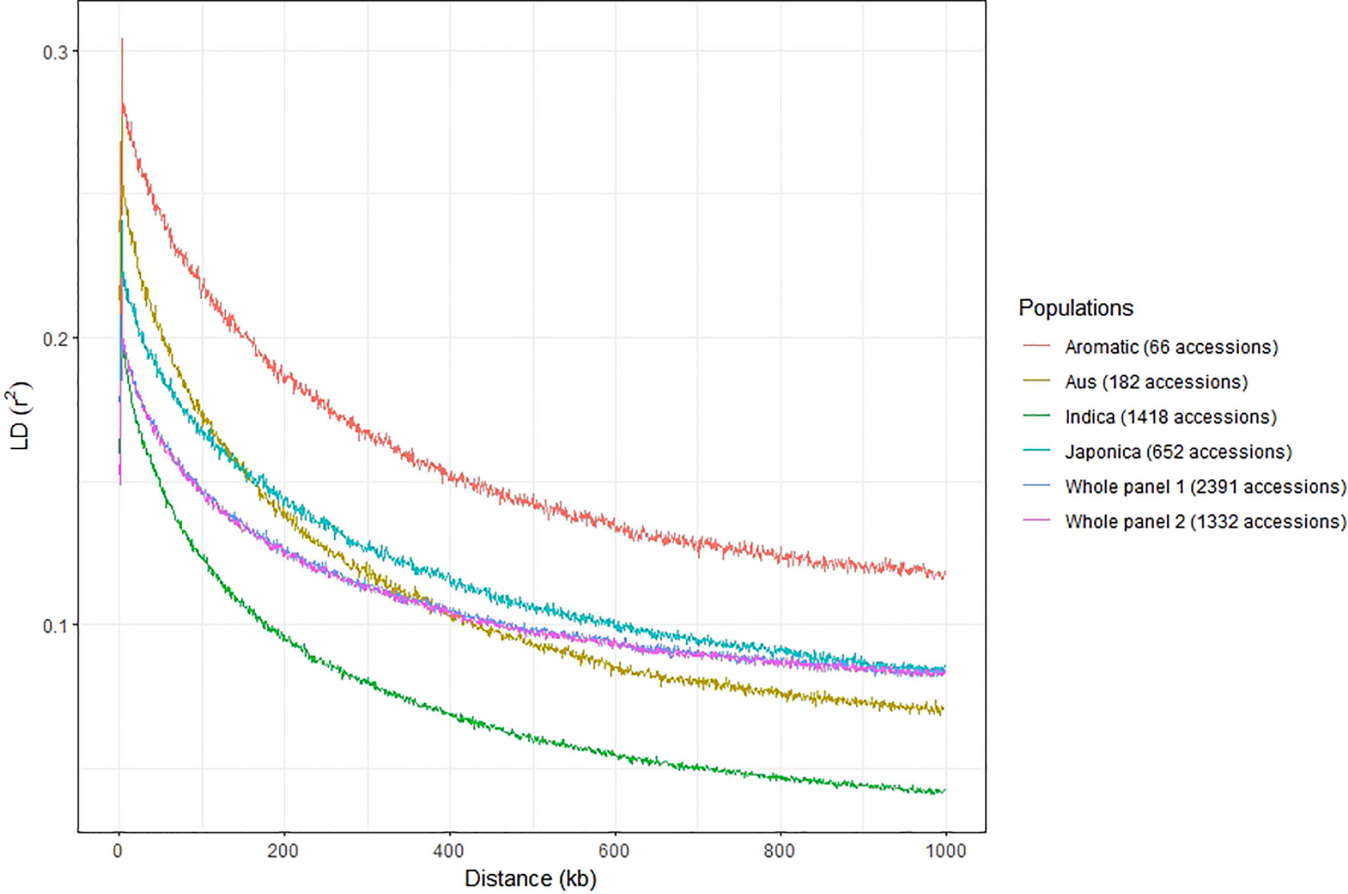
LD decay in whole panel 1 containing 2,391 accessions, whole panel 2 containing 1,332 accessions, and four subspecies *indica, japonica, aus*, and *aromatic* using 117,560 SNPs from the 3K Rice Genomes Project. Whole panels 1 and 2 share the same trend so that the curve for whole panel 1 is partially hidden by the curve of whole panel 2.

LD decay of both whole panels (2,391 and 1,332 accessions) and the four main subgroups (*indica* – 1,418 accessions and *japonica* – 652 accessions, *aus* – 182 accessions, and *aromatic* – 66 accessions from panel 1) were analyzed quickly with 1 kb bin. The results indicated that the LD decay in the *indica* panel occurred on shorter distances than in the *aus*, *japonica*, and *aromatic* panels (**Figure 3**). The LD decayed to its half-maximum within around 134 kb for *indica*, 186 kb for *aus*, 312 kb for *japonica*, 390 kb for *aromatic*, and 280 kb for two whole panels.

### 3.3 Detection of QTLs by GWAS

In this section, we present the results of GWAS of all accessions of both panels (2,391 accessions and 1,332 accessions in the main and confirmatory EXP, respectively) and their subpopulations (1,418 *indica* accessions and 652 *japonica* accessions in the main EXP, and 767 *indica* accessions and 375 *japonica* accessions in the confirmatory EXP). For each trait, we analyzed GWAS with phenotypic data three times: with 2,391 accessions in the main EXP, with 1,332 accessions in the confirmatory EXP, and with the same 1,332 accessions extracted from the 2,391 ones in the main EXP. The Manhattan and Q-Q plot of the GWAS runnings is shown in the **Supplementary Figure S4**. The Q-Q plots for the GWAS results indicated that the model was well-fitted to the data.

We selected the confirmed QTLs, *i.e.* those that were identified in both EXPs. Subsequently, a total of 55 confirmed QTLs for one of the measured traits were detected in each of the four treatments (**Table 2** and **Supplementary Table S4**). These QTLs were named according to the report of McCouch and CGSNL (Committee on Gene Symbolization, Nomenclature and Linkage, Rice Genetics Cooperative) (2008): *qPDWNS, qRDWNS, qSDWNS, qRePDWNS*, (PDWNS, RDWNS, SDWNS, RePDWNS, refers to plant dry weight, root dry weight, shoot dry weight, and the relative plant dry weight between treatments of nitrogen and salt concentrations, respectively) followed by the chromosome number and the detected QTL number in this chromosome. Among these 55 QTLs, 32, 16, and 7 were identified in the whole, the *indica*, and the *japonica* panel, respectively. QTLs were detected neither in all three panels together nor in both *japonica* or *indica* panels. There were some hotspots containing 5 pairs of QTLs detected in both the *indica* and the whole panel, *viz., qPDWNS1.3* and *qPDWNS1.4, qSDWNS1.2* and *qSDWNS1.3, qPDWNS8.2* and *qPDWNS8.3, qRDWNS8.1* and *RDWNS8.2*, and *qSDWNS8.2* and *qSDWNS8.3.* Only one other hotspot contained a pair of QTLs detected in both the *japonica* and the whole panel: *qRePDWNS4.2* and *qRePDWNS4.3.*

Table 2AQTLs were detected for dry weight traits under different N and NaCl treatments in the whole panel in both EXPs.

**Table:** 

No.	*QTL*	Trait	Treatment	Chr.	Peak position	Pvalue	Known genes/QTLs
1	*qPDWNS1.1*	PDW	SN-60Na	1	23013091	1.27E-06	_Na^+^ uptake, K^+^ uptake, ratio Na^+^/K^+^ (Koyama et al., 2001)
							_Root fresh weight (Li et al., 2005)
							_Drought tolerance (Hoang et al., 2019)
2	*qPDWNS1.4*	PDW	SN-0Na	1	29822571	2.73E-05	
3	*qPDWNS3.2*	PDW	SN-0Na	3	33327465	3.66E-06	_Fresh weight, dry weight, plant height, agNUE in low N (Nguyen et al., 2016)
4	*qPDWNS3.4*	PDW	SN-0Na	3	35608381	3.44E-06	_N uptake: *OsAMT3.2* (Suenaga et al., 2003)
							_Length of the third seedling leaf: *qLT3-1* (Cui et al., 2002)
							_1st internode length: *qIN1-3* (Yamamoto et al., 2001)
							_Plant height in low N (Nguyen et al., 2016)
5	*qPDWNS5.1*	PDW	SN-0Na	5	21591128	1.65E-06	_Fresh weight, shoot dry weight in standard N (Nguyen et al., 2016)
6	*qPDWNS7.1*	PDW	SN-0Na	7	6200245	1.13E-05	_Tillers/plant: *tp7b* (Li et al., 2000)
							_Fresh weight, dry weight (Nguyen et al., 2016)
7	*qPDWNS7.2*	PDW	SN-60Na	7	6226107	2.42E-06	_Tillers/plant: *tp7b* (Li et al., 2000)
							_Fresh weight, dry weight (Nguyen et al., 2016)
8	*qPDWNS7.3*	PDW	LN-0Na	7	26352042	6.57E-06	_Root length (Wan et al., 2003)
							_Shoot length: *qSL7* (Jahan et al., 2020)
							_N (Hsieh et al., 2018)
9	*qPDWNS8.2*	PDW	SN-0Na	8	27507063	1.27E-06	_ N (Hsieh et al., 2018)
							_aNUE, agNUE, fresh weight, dry weight (Nguyen et al., 2016)
10	*qPDWNS9.1*	PDW	LN-60Na	9	12272419	1.42E-05	_Salinity tolerance: *OsDREB6* (Ke et al., 2014)
							_Abiotic/biotic tolerance: *OsPAO7* (Liu et al., 2014)
							_Cl^-^ accumulation (Genc et al., 2014)
							_Shoot dry weight (Courtois et al., 2003)
							_Root dry weight: *rdw9* (Li et al., 2005)
							_NUE: *qPFP9.1* (Jewel et al., 2019)
							_Salt susceptible index (Tiwari et al., 2016)
11	*qRDWNS1.2*	RDW	SN-0Na	1	41096834	6.02E-06	_ Plant height in drought (Lafitte et al. 2002) _Biomass plant^-1^: *qBMS1-2* (Hittalmani et al., 2003)
							_Plant height: *ph1* (Yu et al., 2002), *qPH1-1* (Cui et al., 2002)
12	*qRDWNS2.1*	RDW	SN-60Na	2	23177834	2.80E-07	_NUE: *OsNAR2.1* (Yan et al., 2011)
							_NUE: *qPFP1.2* (Jewel et al., 2019)
							_Plant height: *qPH-2* (Mao et al., 2004), *qCSH2* (Han et al., 2007)
							_Root thickness: *qRTT2-1* (Hemamalini et al., 2000)
13	*qRDWNS8.2*	RDW	SN-0Na	8	27640269	6.47E-06	_N (Hsieh et al., 2018)
							_aNUE, agNUE, fresh weight, dry weight (Nguyen et al., 2016)
14	*qRDWNS10.1*	RDW	LN-0Na	10	14391386	7.14E-06	
15	*qRePDWNS2.1*	RePDW	_SN-60Na/SN-0Na_	2	29744672	2.67E-05	
16	*qRePDWNS4.1*	RePDW	_LN-60Na/LN-0Na_	4	1740659	1.69E-06	
17	*qRePDWNS4.2*	RePDW	_LN-60Na/LN-0Na_	4	4298186	1.46E-05	_Salt tolerance *qSKD_4.1* (Batayeva et al., 2018)
18	*qRePDWNS12.1*	RePDW	_SN-60Na/SN-0Na_	12	14854155	9.11E-06	
19	*qRePDWNS12.2*	RePDW	_SN-60Na/SN-0Na_	12	15112248	1.74E-05	_Drought tolerance (Bernier et al., 2007)
20	*qSDWNS1.3*	SDW	SN-0Na	1	29822571	2.08E-05	
21	*qSDWNS3.2*	SDW	SN-0Na	3	33327465	2.56E-06	_Fresh weight, dry weight, plant height, agNUE in low N (Nguyen et al., 2016)
22	*qSDWNS4.1*	SDW	LN-0Na	4	23135184	2.71E-05	_Nitrogen use efficiency *qNUE4.1* (Zhou et al., 2017)
							_Plant height: *qPH1-4-1* (Cui et al., 2004)
23	*qSDWNS5.1*	SDW	SN-0Na	5	21591128	1.58E-06	_Fresh weight, shoot dry weight in standard N (Nguyen et al., 2016)
24	*qSDWNS7.1*	SDW	SN-60Na	7	6307567	9.42E-07	_Tillers/plant: *tp7b* (Li et al., 2000)
							_Fresh weight, dry weight (Nguyen et al., 2016)
25	*qSDWNS7.2*	SDW	LN-0Na	7	20440198	1.83E-06	_Plant height: *Ph7a* (Zenbo et al., 1996)
							_Root dry weight (Wan et al., 2003)
26	*qSDWNS8.2*	SDW	SN-0Na	8	27507063	1.16E-06	_N (Hsieh et al., 2018)
							_aNUE, agNUE, fresh weight, dry weight (Nguyen et al., 2016)
27	*qSRRNS4.1*	SRR	LN-0Na	4	28212797	4.40E-08	
28	*qSRRNS5.1*	SRR	LN-60Na	5	18406533	3.52E-06	
29	*qSRRNS6.1*	SRR	SN-60Na	6	24725704	1.30E-06	_Root to shoot ratio (Ikeda et al., 2006)
30	*qSRRNS7.1*	SRR	SN-60Na	7	20705889	1.57E-05	
31	*qSRRNS7.1*	SRR	LN-60Na	9	1602896	7.57E-06	
32	*qSRRNS12.1*	SRR	LN-0Na	12	3395136	4.46E-07	

Chr, Chromosome.

**Table 2B:** QTLs were detected for dry weight traits under different N and NaCl treatments in the *indica* panel in both EXPs.

No.	*QTL*	Trait	Treatment	Chr.	Peak position	Pvalue	Known genes/QTLs
1	*qPDWNS1.2*	PDW	SN-60Na	1	29517723	1.27E-06	
2	*qPDWNS1.3*	PDW	SN-0Na	1	29612773	1.16E-05	
3	*qPDWNS1.5*	PDW	SN-60Na	1	33808595	1.46E-05	
4	*qPDWNS2.1*	PDW	SN-0Na	2	29617039	1.60E-06	_Seedling dry weight: *qSDW2* (Han et al., 2007)
							_Plant height: *qPH-2* (Mao et al., 2004), in standard N (Nguyen et al., 2016)
5	*qPDWNS3.3*	PDW	LN-0Na	3	35495246	2.14E-05	_N uptake: *OsAMT3.2* (Suenaga et al., 2003)
							_Length of the third seedling leaf: *qLT3-1* (Cui et al., 2002)
							_1st internode length: *qIN1-3* (Yamamoto et al., 2001)
							_Plant height in low N (Nguyen et al., 2016)
6	*qPDWNS8.3*	PDW	SN-0Na	8	27602390	1.43E-05	_N (Hsieh et al., 2018)
							_aNUE, agNUE, fresh weight, dry weight (Nguyen et al., 2016)
7	*qRDWNS1.1*	RDW	SN-60Na	1	29517723	2.94E-05	
8	*qRDWNS4.1*	RDW	LN-60Na	4	19933152	1.72E-07	
9	*qRDWNS8.1*	RDW	SN-0Na	8	27602390	5.92E-06	_N (Hsieh et al., 2018)
							_aNUE, agNUE, fresh weight, dry weight (Nguyen et al., 2016)
10	*qRePDWNS3.1*	RePDW	_LN-60Na/SN-60Na_	3	25687022	2.84E-10	
11	*qSDWNS1.1*	SDW	SN-60Na	1	29517723	8.08E-07	
12	*qSDWNS1.2*	SDW	SN-0Na	1	29612773	1.04E-05	
13	*qSDWNS1.4*	SDW	SN-0Na	1	30991676	7.13E-06	
14	*qSDWNS1.5*	SDW	SN-60Na	1	33808595	1.83E-05	
15	*qSDWNS1.6*	SDW	SN-0Na	1	37614990	1.34E-05	
16	*qSDWNS8.3*	SDW	SN-0Na	8	27602390	2.47E-05	_N (Hsieh et al., 2018)
							_aNUE, agNUE, fresh weight, dry weight (Nguyen et al., 2016)

Chr, Chromosome.

**Table 2C:** QTLs were detected for dry weight traits under different N and NaCl treatments in the *japonica* panel in both EXPs.

No.	*QTL*	Trait	Treatment	Chr.	Peak position	Pvalue	Known genes/QTLs
1	*qPDWNS3.1*	PDW	SN-0Na	3	4974560	1.32E-06	
2	*qPDWNS8.1*	PDW	SN-0Na	8	573918	1.50E-06	
3	*qRePDWNS4.3*	RePDW	_LN-60Na/LN-0Na_	4	4403318	1.56E-06	_Salt tolerance *qSKD_4.1* (Batayeva et al., 2018)
4	*qRePDWNS8.1*	RePDW	_LN-60Na/LN-0Na_	8	26569164	7.25E-06	
5	*qSDWNS3.1*	SDW	SN-0Na	3	4974560	7.85E-06	
6	*qSDWNS8.1*	SDW	SN-0Na	8	573918	2.98E-06	
7	*qSDWNS10.1*	SDW	LN-0Na	10	18157987	8.57E-07	

Chr, Chromosome.

More QTLs (24) were detected under standard conditions (SN-0Na) than in the three other treatments (8, 11, and 4 QTLs under LN-0Na, SN-60Na, and LN-60Na, respectively). Among the 32 QTLs detected in the whole panel, 12 were found in SN-0Na, 6 in LN-0Na, 6 in SN-60Na, 3 in LN-60Na, and 5 for the relative PDW between treatments. In the *indica* panel, the number of QTLs detected for these five treatments and relative PDW was 8, 1, 5, and 1, respectively, while in the *japonica* there were 4, 1, 0, and 0, respectively. One QTL for relative PDW between salinity and non-salinity under standard N was identified in the *indica* panel, and 1 QTL for the relative PDW between low and standard N under saline treatment was detected in the *japonica* panel. None of the QTLs was detected in all four treatments simultaneously. Three QTLs were detected in SN-60Na overlapped with three QTLs detected in SN-0Na: *qPDWNS1.2, qSDWNS1.1, qPDWNS7.2* overlapped with *qPDWNS1.3, qSDWNS1.2*, and *qPDWNS7.1*, respectively. Another QTL *qPDWNS3.4* in SN-0Na was detected in an overlapping region containing *qPDWNS3.3* detected in LN-0Na.

### 3.4 Candidate genes for important QTLs

**Figure 4:**
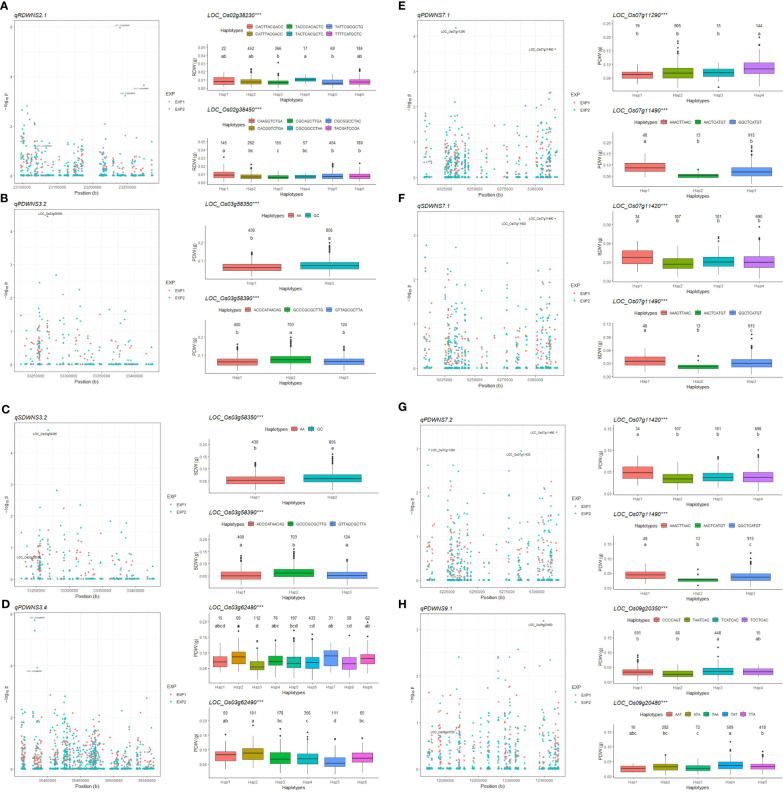
Gene-based association and haplotype analysis of targeted genes for *qRDWNS2.1* **(A)**, *qPDWNS3.2* **(B)**, *qSDW3.2* **(C)**, *qPDWNS3.4* **(D)**, *qPDWNS7.1* **(E)**, *qSDWNS7.1* **(F)**, *qPDWNS7.2* **(G)** and *qPDWNS9.1* **(H)**. Each point is one SNP in the association of the QTLs. The value and letter on the boxplot (a, b, c, and d) indicate the number of individuals in each haplotype in the confirmatory EXP and multiple comparison results at the significance level of 0.05, respectively. *** significance level of ANOVA for phenotypic value of the main haplotypes at p < 0.001.

Among the 55 QTLs, we focused on some important QTLs in the whole panel – *i.e.* either with a lot of SNPs that passed the threshold or close to previously reported cloned genes or fine-mapped QTLs – to identify candidate genes. Thus, we detected 12 important QTLs, *viz. qRDWNS2.1, qPDWNS3.2, qSDWNS3.2, qPDWNS3.4, qPDWNS7.1, qSDWNS7.1, qPDWNS7.2, qPDWNS7.3, qPDWNS8.2, qRDWNS8.2, qSDWNS8.2*, and *qPDWNS9.1* that we retained for the further step of identifying candidate genes. Afterward, we were able to narrow down to a relatively small number of candidate genes, using a resolution of 200 kb for all identified important QTLs, resulting in 242 genes (**Supplementary Table S6**). Subsequently, we re-analyzed GWAS for the target traits by gene-based analysis of each QTL by multi-locus mrMLM (Zhang, 2019). Finally, matching SNPs passing the threshold in the gene-based analysis of the 242 genes above allowed us to select 12 genes harboring SNPs passing the threshold – -log_10_p-value ≥ 3 – to test significant phenotypic differences between haplotypes (**Supplementary Table S7**). Apart from the 12 genes above, we selected 3 additional genes based on their functional annotation in the database (Kawahara et al., 2013) for haplotype analysis (**Supplementary Table S6**). The values of the target traits for the different haplotypes in each of the 15 genes were analyzed through ANOVA. Significant differences were found among the major haplotypes in 11 of the 15 genes, which could be considered promising candidates associated with the target traits (**Table 3**; **Figure 4**; **Supplementary Table S8**). Meanwhile, 8 candidate genes were shortlisted from the 12 genes that passed the threshold (-log_10_p-value ≥ 3), and 3 other candidate genes that did not pass the threshold were identified from the selected genes in the annotation. Among them, six candidate genes of three important QTLs related to DW under SN-0Na were shortlisted, *viz. LOC_Os03g58350* and *LOC_Os03g58390* in *qPDWNS3.2* and *qSDWNS3.2; LOC_Os03g62480* and *LOC_Os03g62490* in *qPDWNS3.4; LOC_Os07g11290* and *LOC_Os07g11490* in *qPDWNS7.1*. Under SN-60Na, four candidate genes of three QTLs were detected, *viz. LOC_Os02g38230* and *LOC_Os02g38450* in *qRDWNS2.1, LOC_Os07g11420* and *LOC_Os07g11490* for *qSDWNS7.1* and *qPDWNS7.2.* Under LN-60Na, two candidate genes were detected in *qPDWNS9.1*: *LOC_Os09g20350* and *LOC_Os09g20480*. Finally, no candidate gene related to DW in LN-0Na was selected.

**Table 3:** List of 11 candidate genes for the important QTLs identified related to nitrogen use efficiency at the seedling stage under four N and NaCl treatments.

No	*Genes*	Chr.	Gene Product Name	Trait	Treatment	*QTL*
1	*LOC_Os02g38230*	2	high-affinity nitrate transporter, putative, expressed	RDW	SN-60Na	*qRDWNS2.1*
2	*LOC_Os02g38450*	2	expressed protein	RDW	SN-60Na	*qRDWNS2.1*
3	*LOC_Os03g58350*	3	*OsIAA14* - Auxin-responsive Aux/IAA gene family member, expressed	PDW, SDW	SN-0Na	*qPDWNS3.2, qSDWNS3.2*
4	*LOC_Os03g58390*	3	zinc finger, C3HC4 type domain containing protein, expressed	PDW, SDW	SN-0Na	*qPDWNS3.2, qSDWNS3.2*
5	*LOC_Os03g62480*	3	anthocyanidin 5,3-O-glucosyltransferase, putative, expressed	PDW	SN-0Na	*qPDWNS3.4*
6	*LOC_Os03g62490*	3	prohibitin-2, putative, expressed	PDW	SN-0Na	*qPDWNS3.4*
7	*LOC_Os07g11290*	7	expressed protein	PDW	SN-0Na	*qPDWNS7.1*
8	*LOC_Os07g11420*	7	transposon protein, putative, CACTA, En/Spm sub-class	PDW, SDW	SN-60Na	*qPDWNS7.2, qSDWNS7.1*
9	*LOC_Os07g11490*	7	expressed protein	PDW, SDW	SN-0Na, SN-60Na	*qPDWNS7.1, qSDWNS7.1, qPDWNS7.2*
10	*LOC_Os09g20350*	9	ethylene-responsive transcription factor, putative, expressed	PDW	LN-60Na	*qPDWNS9.1*
11	*LOC_Os09g20480*	9	transporter, putative, expressed	PDW	LN-60Na	*qPDWNS9.1*

Chr, chromosome.

Based on the haplotype analysis and candidate genes identification, many useful accessions with high dry weight were shortlisted for future functional characterization related to NUE under saline condition. The accessions with haplotype ‘CATTTACGACC’ in *LOC_Os02g38230 (OsNAR2.1)* gene showed high RDW, e.g. Dawk Put, Nona Bokra, Ex Ebokozuru, Aus 78-125, Tsimatahopaosa, among other genotypes. The accessions with haplotype ‘AGGTGATAGAGCACCAAGAGGAGGAGGCCAAATATAA’ in *LOC_Os07g11420* and haplotype ‘AAACTTAAC’ in *LOC_Os07g11490* produced higher SDW and PDW than the accessions containing other haplotypes. These genotypes were – among others – Dawk Put, Do Khaw, Aus 278, Doc Phung D12, LG 9274, Khao Hae in *LOC_Os07g11420* and Khie Tom, Patnai 31-679, Kyauk Kyi, Suga Pankha, Quahng Luang, Toun, Pokkali, ARC 14737 in *LOC_Os07g11490*. The accessions with haplotype ‘TCATCAC’ in *OsDREB6 (LOC_Os09g20350)* gene showed higher PDW under LN-60Na conditions, e.g. Nona Bokra, Matali, Sachi, Mestre, RD 19, Babaomi, Sipot, San Bao Gu, Doc Phung D12 (**Supplementary Table S9**).

## 4 Discussion

This study investigated the dry weights of rice grown under different conditions of N and NaCl concentrations. The results showed that SDW decreased with either N-deficiency or NaCl application. It may be linked to a reduction in chlorophyll content, N uptake, N content, and photosynthetic capacity (Abdelgadir et al., 2005; Song et al., 2019; Phan et al., 2023). Moreover, under N-deficiency, rice plants do not have enough energy to maintain antioxidant activities to cope with oxidative damage, leading to over-accumulation of Na^+^ and decrease in growth under saline conditions (Chen et al., 2022; Phan et al., 2023). RDW, however, was reduced by the presence of NaCl but increased under N-deficiency treatment. On the one hand, RDW may be reduced by salt by reducing root number, altering root morphological characteristics, as well as root oxidation capacity (Chen et al., 2022; Phan et al., 2023). On the other hand, RDW may be enhanced by decreasing N because N-deficiency promotes root length by enhancing cell division, cell elongation, amount of auxin, or by modifying the interaction between auxin and abscisic acid (Hsieh et al., 2018). The effect of N and NaCl also resulted in changes in phenotypic variation in SDW and RDW: with the largest variation in SN-0Na, following SN-60Na, LN-0Na, and the lowest in LN-60Na (**Figure 3** and **Table 1**). The change in the phenotypic variation also might lead to a difference in the number of QTLs detected under different N and NaCl conditions. Indeed, SN-0Na treatment allowed to detect a very large number of QTLs (16), followed by SN-60Na (11), LN-0Na (8), and finally LN-60Na (4).

LD decay occurred more quickly in the *indica* panel, followed by *aus*, *japonica*, and finally in the *aromatic* panel. It was consistent with previous studies. Shi et al. (2017) also showed that LD decayed more quickly in the *indica* group than in non-*indica* groups. Mather et al. (2007) indicated that LD extends over a shorter distance in *indica* than *tropical japonica* and *temperate japonica*. The differences between the LDs among the groups were linked to differences in outcrossing and recombination rate. Higher recombination rates were associated with lower LD.

QTL identification can be influenced by both genotype-environment interactions and by the number of accessions in the population. Norton et al. (2021) and Talukdar et al. (2022) detected different QTLs in different years of research or different irrigated systems and none of the QTLs were detected under all the conditions. In our research, two EXPs were conducted with different numbers of accessions and light competition among the accessions. In the entire panel, we detected 157 loci for the tested traits from 2,391 accessions in the main EXP and 138 loci from 1,332 accessions in the confirmatory EXP (**Supplementary Table S3A**). Among them, 32 common QTLs were considered reliable because they were detected in both EXPs. Previously, Batayeva et al. (2018) detected two QTLs related to dry weight in non-saline condition but none of them under saline conditions by using a population containing 176 *temperate japonica* accessions. In the current study, we detected 3 of 32 QTLs under both saline and non-saline condition, with standard N supply. They co-localized with previously reported QTLs for tiller number per plant, fresh and dry weight of plants (Li et al., 2000; Nguyen et al., 2016). Other QTLs for DWs identified in our study under only saline but not under non-saline treatments were also found in the genomic regions containing QTLs for salt tolerance, *viz.* Na^+^ uptake, K^+^ uptake, and Na^+^/K^+^ concentration in the tissue (Koyama et al., 2001), drought tolerance (Hoang et al., 2019), salt susceptibility index (Tiwari et al., 2016), and Cl^-^ accumulation (Genc et al., 2014; Ke et al., 2014; Liu et al., 2014). Phan et al. (2023) reported that the salt-tolerant cultivars performed high NUE because they accumulated less Na^+^ than the sensitive ones. Therefore, under saline conditions, the plants accumulating less toxic ions such as Na^+^ and Cl^-^ could maintain water and nitrogen uptake. Consequently, the absorbed N can be better assimilated, resulting in higher tiller number, and finally, growth could be maintained. Among the 55 detected QTLs detected whether in the entire (32), or *indica* (16), or *japonica* panel (7) and confirmed in both EXPs in the present study, there were 28 QTLs for NUE-related traits which had never been reported before, *i.e* the QTLs located on chromosome 1, 2, 3, 5, 8, 10, and 12. The 27 other ones co-localized with previously reported QTLs for NUE-related traits (**Table 2**). Interestingly, a lot of QTLs co-localized with different traits in various studies. Indeed, *qPDWNS3.3* and *qPDWNS3.4* co-localized with the QTLs for the length of the first internode, length of the third leaf, plant height, and N uptake *OsAMT3.2* (Yamamoto et al., 2001; Cui et al., 2002; Li et al., 2012; Suenaga et al., 2003; Nguyen et al., 2016). *OsAMT3.2* is one of the ammonium transporter genes regulating ammonium uptake, expressed in old leaves, and was not influenced by salt stress (Wang et al., 2012). *qPDWNS7.3* in the region containing QTLs for root length, shoot length, and N metabolism (Wan et al., 2003; Hsieh et al., 2018; Jahan et al., 2020). *qRDWNS2.1* for RDW detected in SN-60Na was in an interval containing *OsNAR2.1* and *qPFP1.2* for NUE and in the region containing a QTL for root thickness (Hemamalini et al., 2000), and plant height (Mao et al., 2004; Han et al., 2007). *OsNAR2.1* regulates N uptake and is related to drought tolerance (Yan et al., 2011; Fan et al. 2017; Chen et al., 2019). *qPDWNS9.1* detected in the LN-60Na treatment has been related to dry weight and salinity tolerance in various studies (Courtois et al., 2003; Li et al., 2005; Genc et al., 2014; Ke et al., 2014; Liu et al., 2014; Tiwari et al., 2016; Jewel et al., 2019). This *qPDWNS9.1* region contained *OsDREB6* gene which was reported playing an important role in enhancing tolerance to osmotic, salinity, and cold stress (Ke et al., 2014).

Reanalyzing by multi-locus GWAS in mrMLM package plus searching genes with functional annotation and haplotype analysis allowed us to identify 11 candidate genes for SDW, RDW, and PDW in eight important QTLs under SN-0Na, SN-60Na, and LN-60Na (**Table 3**). Six, four, and two candidates were detected in SN-0Na, SN-60Na, and LN-60Na treatments, respectively. Among them, one gene was detected for PDW in both SN-0Na and SN-60Na treatments. In SN-0Na (normal condition), the six candidate genes detected in this study differed from the two QTLs identified previously (Batayeva et al., 2018). Among these six candidate genes, *LOC_Os03g58350* (*OsIAA14*) is a gene belonging to the auxin-responsive Aux/IAA gene family, that regulates lateral root development in rice *via* auxin signaling (Zhang et al., 2018). *LOC_Os03g58390 (OsSIRP2)* has been associated with salinity tolerance and osmotic tolerance (Chapagain et al., 2018). The other candidate genes were *LOC_Os03g62480*, *LOC_Os03g62490*, *LOC_Os07g11290*, and *LOC_Os07g11490*.

Under the SN-60Na condition, four candidate genes were identified: two candidate genes for RDW on chromosome 2 and two candidates for SDW and PDW together on chromosome 7. The two candidate genes for *qRDWNS2.1* controlling RDW in SN-60Na on chromosome 2: *LOC_Os02g38230 (OsNAR2.1)* and *LOC_Os02g38450*. Overexpression of protein OsNAR2.1 improves N uptake as well as chlorophyll content, photosynthetic rate, water use efficiency, and grain yield under drought stress (Chen et al., 2019) but no information related to *OsNAR2.1* in rice has been reported under saline conditions. This gene interacts with *OsNRT2.1, OsNRT2.2*, and *OsNRT2.3a* and plays a key role in enabling plants to cope with a variable nitrate supply (Yan et al., 2011). Wang et al., (2012) reported that the expression level of the *OsNRT* gene family was influenced by salt stress, thereby reducing nitrate accumulation in salt conditions. Especially, Shi et al. (2017) also documented that the gene *OsNRT2.2*, which interacts with *OsNAR2.1*, was associated with the salt susceptibility index in rice at the germination stage. In our study, we found that *OsNAR2.1* is a candidate for controlling RDW under saline condition. Another candidate gene for *qRDWNS2.1* was *LOC_Os02g38450* for which no gene ontology classification has been published. Other candidate genes on chromosome 7, viz. *LOC_Os07g11420* and *LOC_Os07g11490* for *qPDWNS7.1*, *qSDWNS7.1*, and *qPDWNS7.2*. were poorly documented previously.

In LN-60Na treatment, *OsDREB6 (LOC_Os09g20350)* in *qPDWNS9.1* is an ethylene-responsive transcription factor that controls tolerance to osmotic, drought, cold, and salinity stresses (Ke et al., 2014). Some varieties with ‘TCATCAC’ haplotype, such as Nona Bokra, Doc Phung D12 have been well-documented for their salt tolerance (Lutts et al., 1996; Ho et al., 2018). Another candidate gene for QTL *qPDWNS9.1*, *LOC_Os09g20480* (homolog to *Sb02g023340* in sorghum and *Traes_5AL_F8B48EC59* in wheat), encodes a transporter protein. This gene has been reported to be related to Cl^-^ accumulation in wheat (Genc et al., 2014).

## 5 Conclusion

By realizing a GWAS on 2,391 accessions with 235,210 SNPs and confirming the results on 1,332 accessions, we detected 55 QTLs for SDW, RDW, PDW, and relative PDW in different treatments. Among them, 28 QTLs were novel and the other 27 QTLs co-located with previously detected ones. Three of 11 QTLs that were identified under salt treatments were close to regions containing 3 QTLs detected in non-saline conditions. Some of the detected QTLs for DWs under saline conditions co-localized with known QTLs or genes for salt tolerance. Then further haplotype analysis allowed us to identify 11 candidate genes for eight important QTLs related to DW traits. Further study should be carried out to validate these genes in field environment. Moreover, salt and N concentrations in the tissue as well as NUE components should be determined and submitted to GWAS to find genetic information as well as candidate genes associated with these traits. Our results provide useful germplasm and genetic information for the future improvement of NUE in rice and rice production.

## Data availability statement

The datasets presented in this study can be found in online repositories. The names of the repository or repositories and accession number(s) can be found in the article or **Supplementary Material**.

## Author contributions

NP, CP, and PB designed the project. NP analyzed the GWAS and identified the candidate genes. NP, XD, and PB wrote and revised the manuscript. All authors read and approved the final manuscript.
