# Supplementary figures and images for: Identification of quantitative trait loci controlling nitrogen use efficiency-related traits in rice at the seedling stage under salt condition by genome-wide association study
Source: Front Plant Sci. 2023 Jul 27;14:1197271. doi: 10.3389/fpls.2023.1197271 (PMC10415682; doi:10.3389/fpls.2023.1197271)

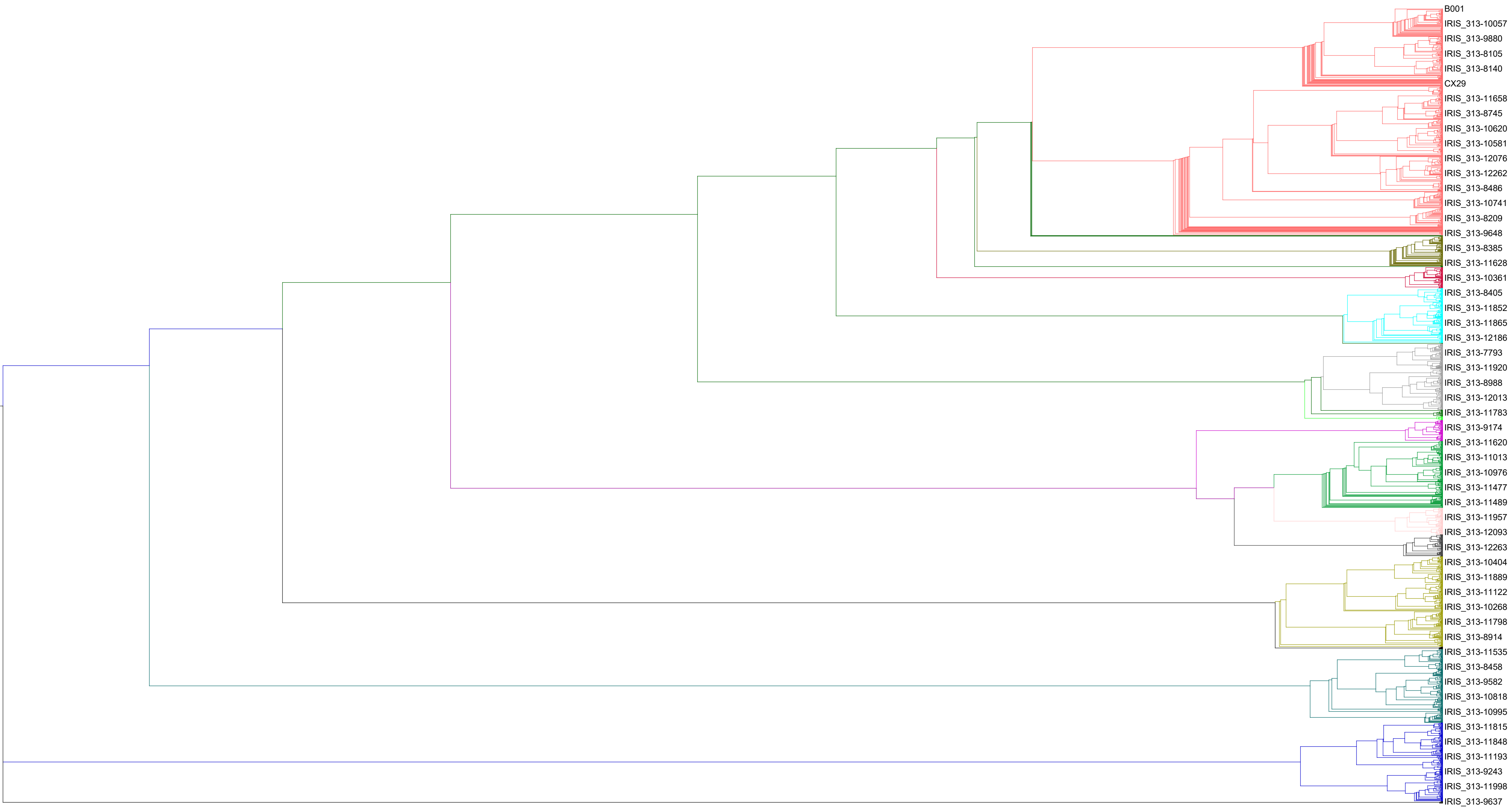

Supplement: Supplementary file 1 [file DataSheet_1.pdf]
