# Supplementary material for: Identification of quantitative trait loci controlling nitrogen use efficiency-related traits in rice at the seedling stage under salt condition by genome-wide association study
Source: Front Plant Sci. 2023 Jul 27;14:1197271. doi: 10.3389/fpls.2023.1197271 (PMC10415682; doi:10.3389/fpls.2023.1197271)
Supplement: Supplementary file 2 [file DataSheet_2.pdf]

Supplement for manuscript entitle "Identification of quantitative trait loci controlling nitrogen use efficiency-related traits in rice at the seedling stage under salt condition by genome-wide association study"

Fig. S2, S3, and S4

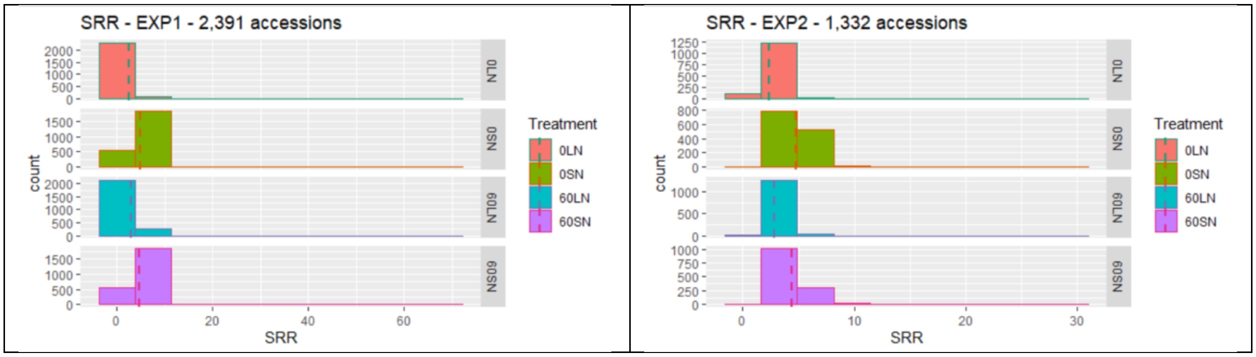

Fig S2. Phenotypic variation of SDW, RDW, PDW, and SRR traits in four treatments in two experiments. 0LN: no NaCl adding and low N concentration, 0SN: no NaCl adding and standard N concentration, 60LN: 60 mM NaCl adding and low N concentration, 60SN: 60 mM NaCl adding and standard N concentration

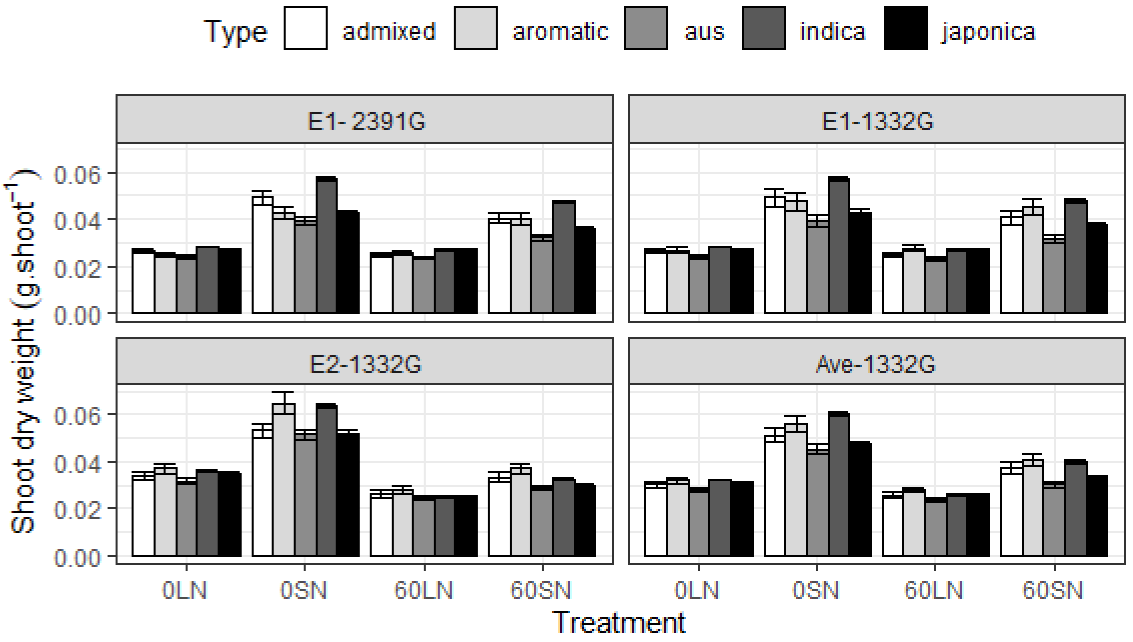

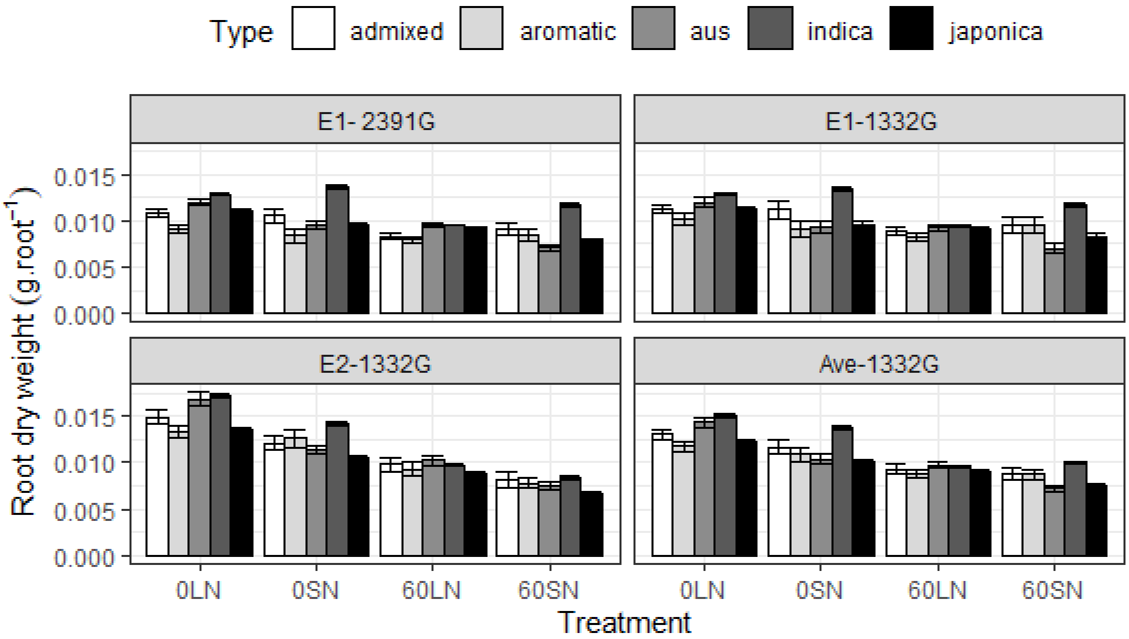
Type admixed aromatic aus indica japonica
E1- 2391G
E1-1332G
E2-1332G
Ave-1332G
Root dry weight (g.root^{-1})
0.000
0.005
0.010
0.015
0LN
0SN
60LN
60SN
Treatment

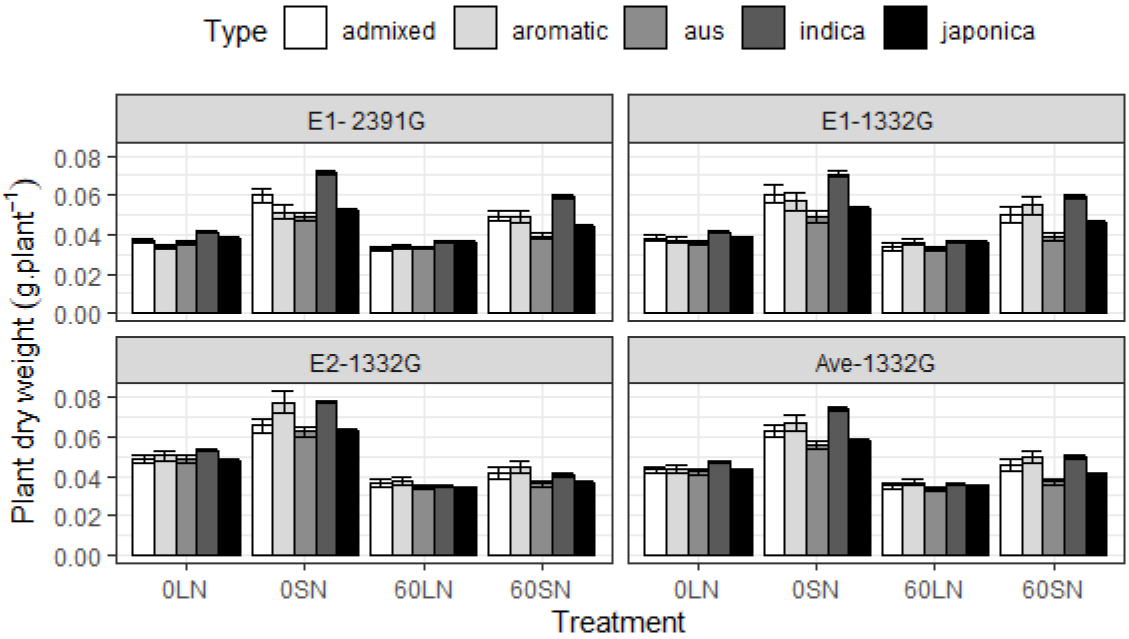
Type admixed aromatic aus indica japonica
E1- 2391G
E1-1332G
E2-1332G
Ave-1332G
Plant dry weight (g.plant^{-1})
0.00
0.02
0.04
0.06
0.08
0LN
0SN
60LN
60SN
Treatment

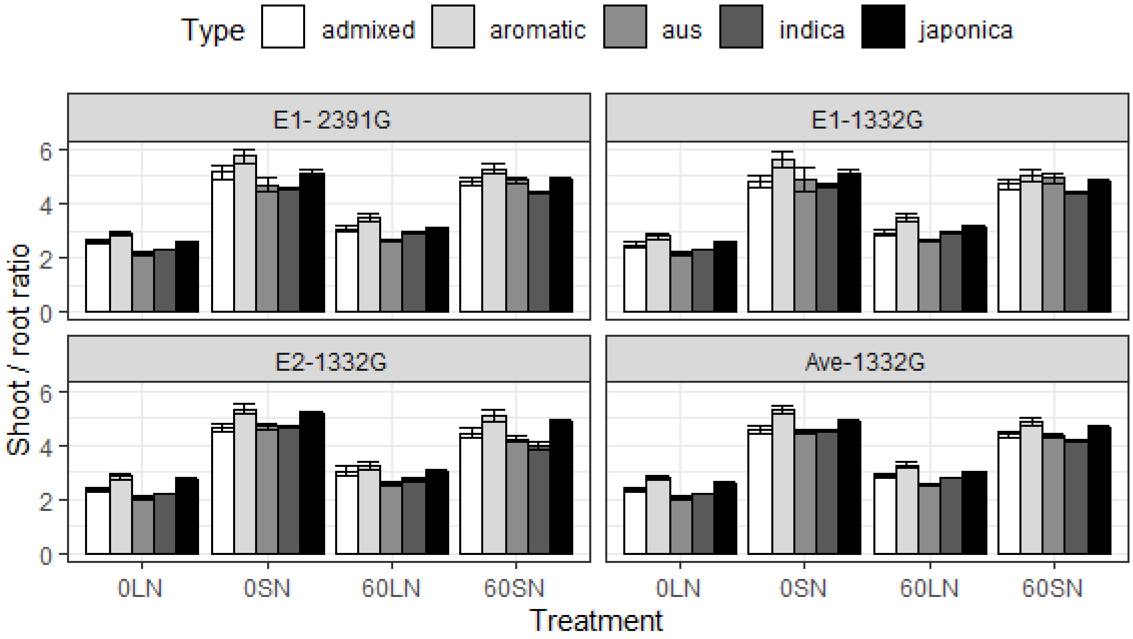

Fig. S3 Shoot dry weight, root dry weight, plant dry weight, and shoot and root ratio of different subgroups in different conditions of N and NaCl concentrations

Manhattan plot and Q-Q plot of PDW, SDW, and RDW in two experiments

1. Treatment of 0 NaCl x standard N (0SN)

   1.1. Plant dry weight (PDW)

EXP1 - 2391	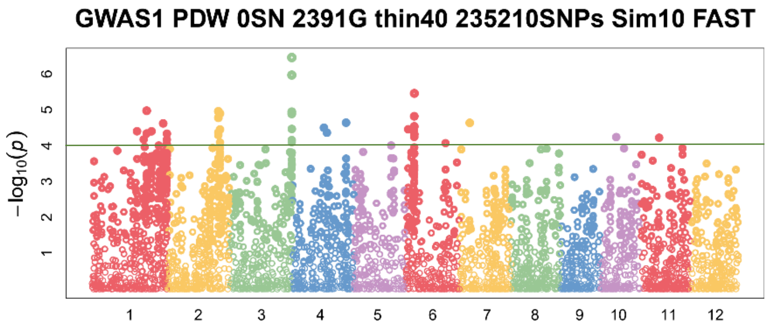	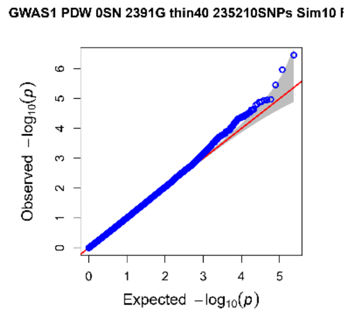
EXP1 - 1332	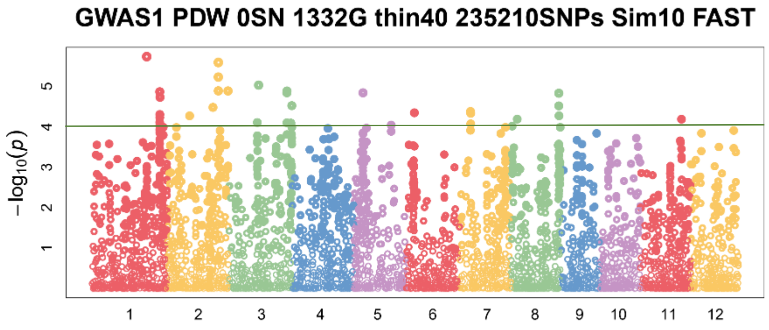	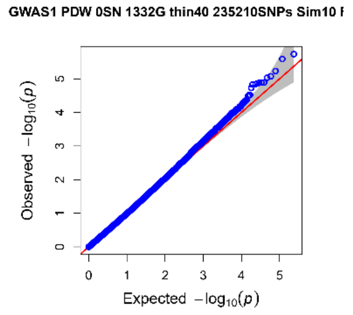
EXP2 - 1332	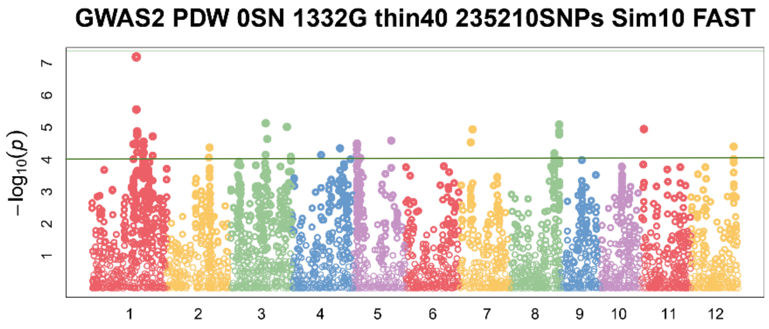	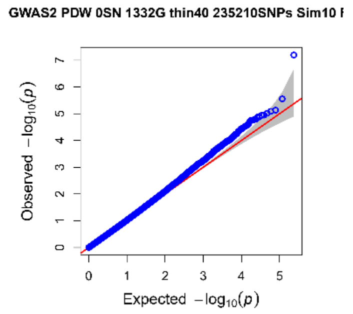

   1.2. Shoot dry weight (SDW)

<table>
<tr><td>EXP1-
2391</td><td>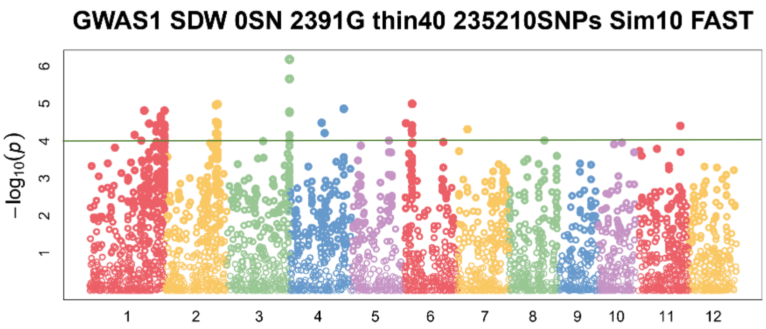
</td><td>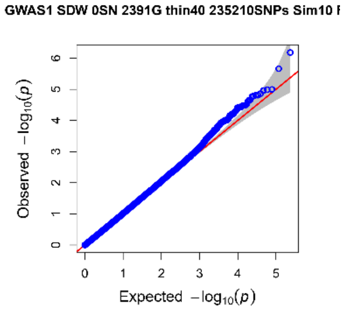
</td></tr>
<tr><td>EXP1-
1332</td><td>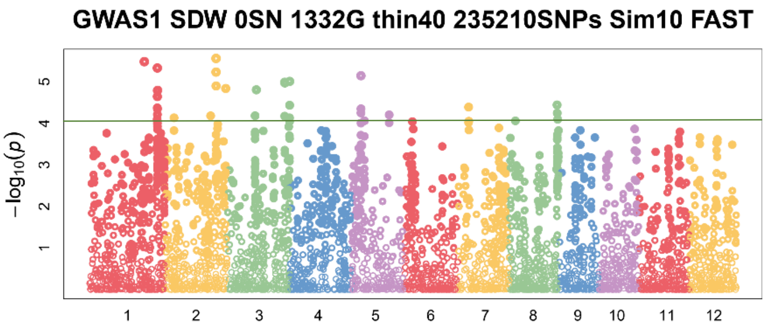
</td><td>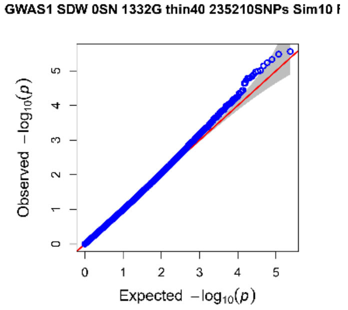
</td></tr>
<tr><td>EXP2-
1332</td><td>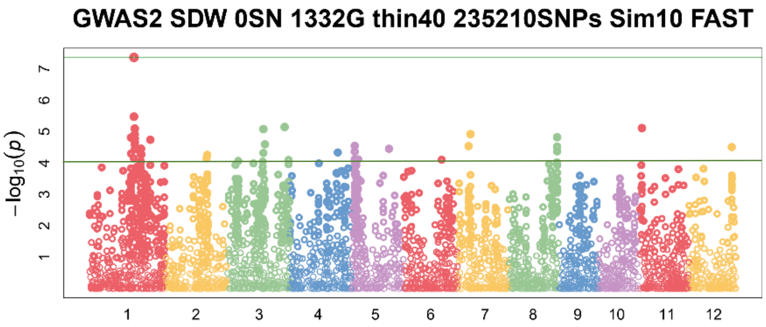
</td><td>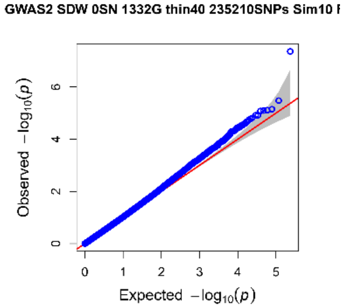
</td></tr>
</table>

1.3. Root dry weight (RDW)

<table>
<tr><td>EXP1-
2391</td><td>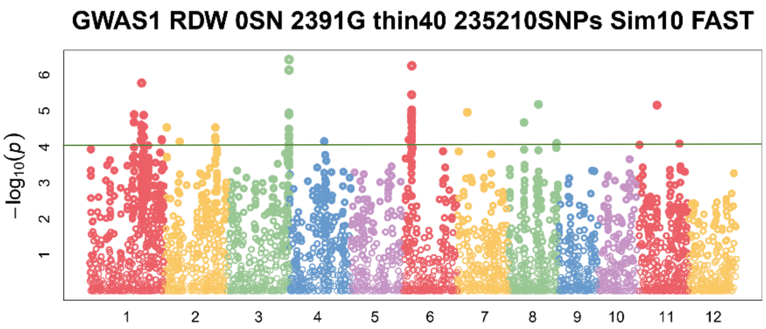
</td><td>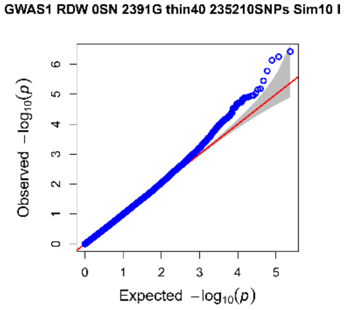
</td></tr>
</table>

EXP1-1332

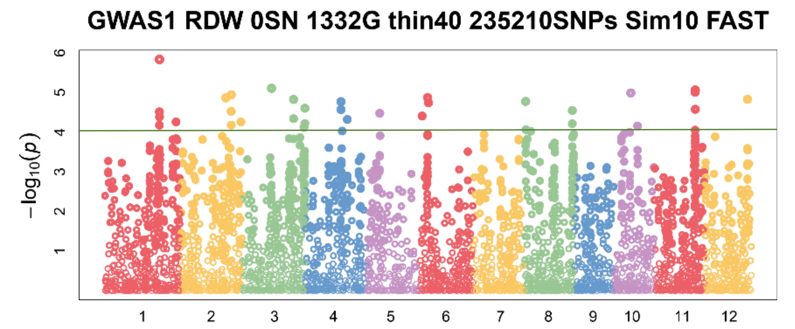

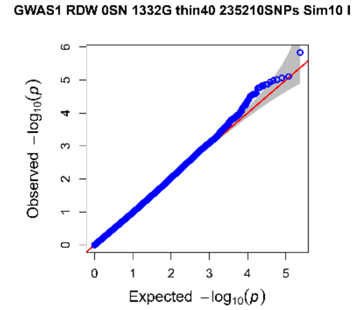

EXP2-1332

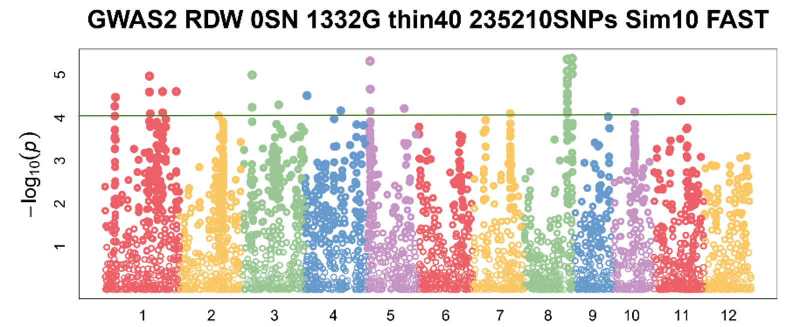

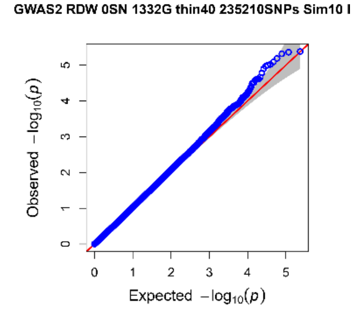

1.4. Ratio of shoot dry weight and root dry weight

EXP1-2391

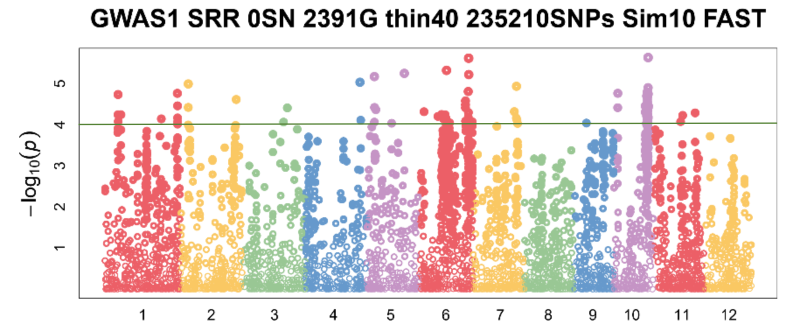

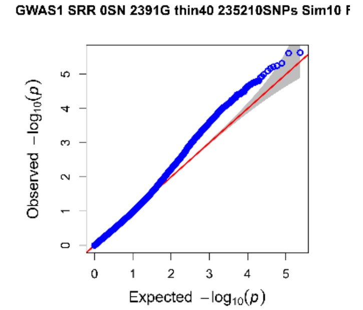

EXP1-1332

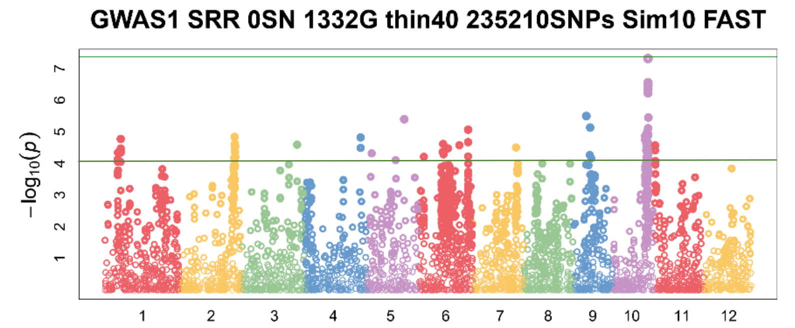

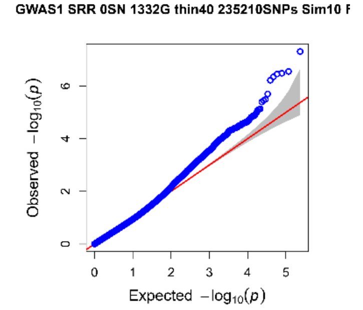

EXP2-1332	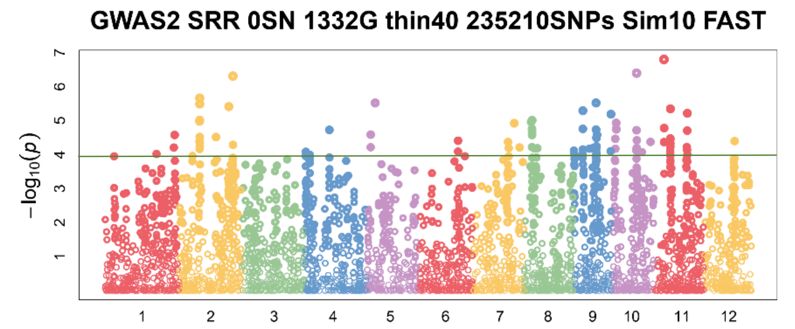GWAS2 SRR 0SN 1332G thin40 235210SNPs Sim10 FAST<br>−log10(p)<br>1 2 3 4 5 6 7 8 9 10 11 12	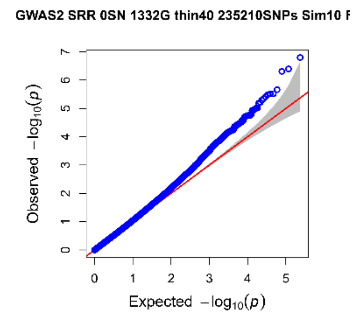GWAS2 SRR 0SN 1332G thin40 235210SNPs Sim10 F<br>Observed −log10(p)<br>Expected −log10(p)

2. Treatment of 0 NaCl x low N (LN-60Na)

2.1. Plant dry weight (PDW)

EXP1-2391	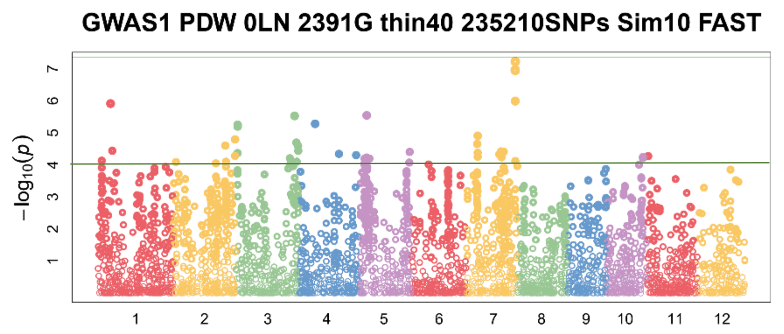	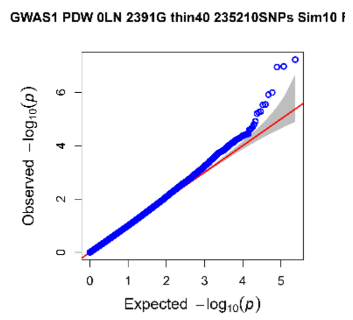
EXP1-1332	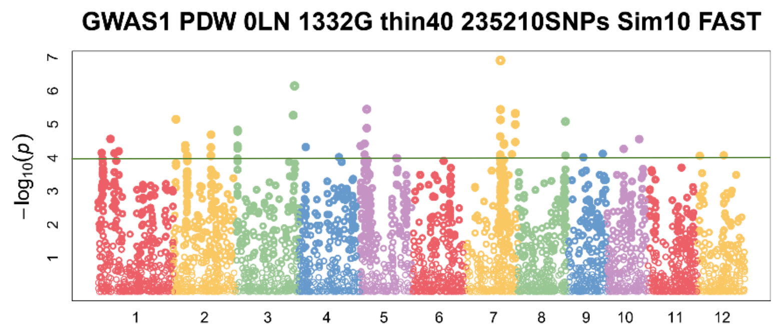	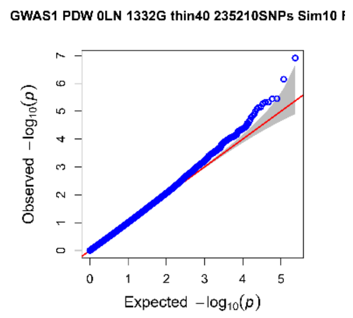
EXP2-1332	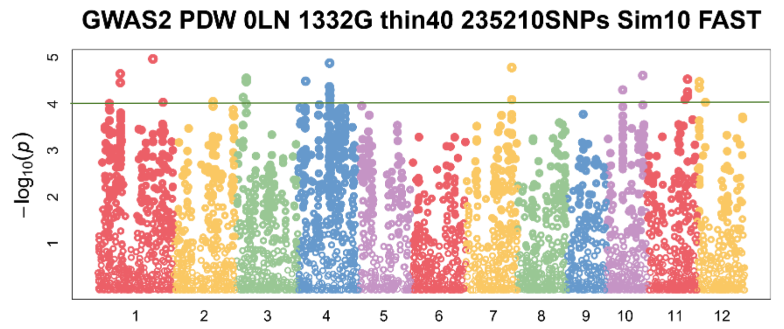	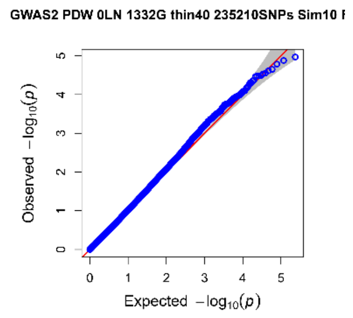

2.2. Shoot dry weight (SDW)

EXP1-2391	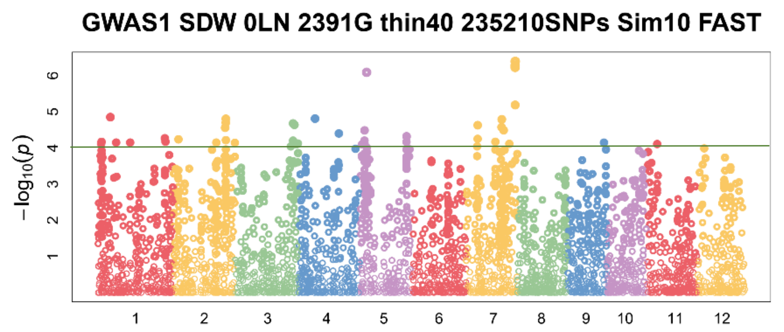	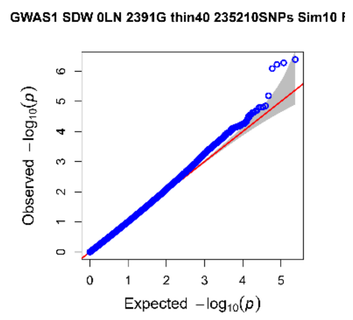

EXP1-1332

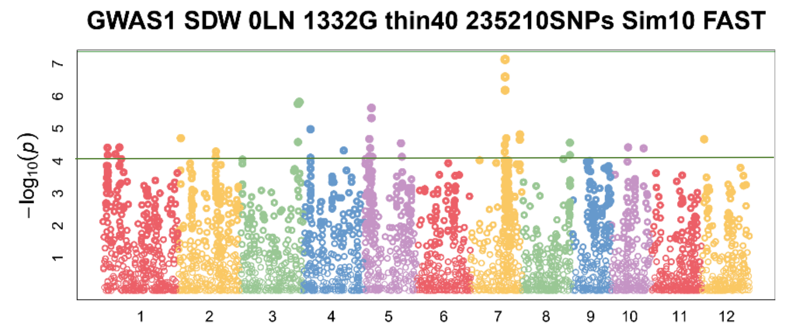

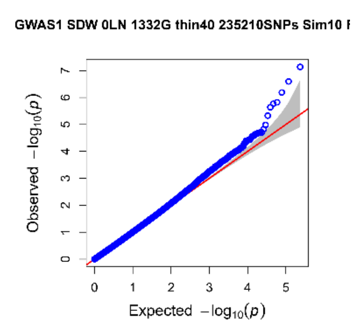

EXP2-1332

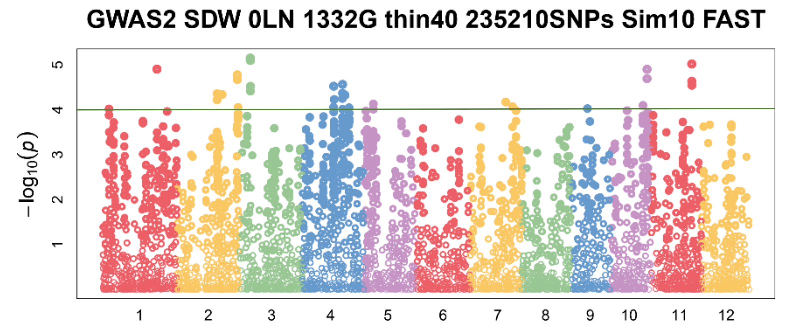

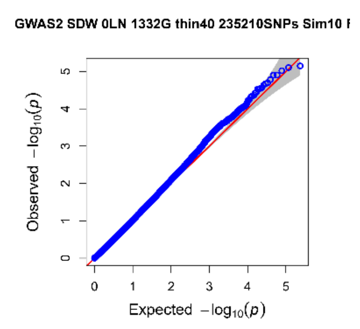

2.3. Root dry weight (RDW)

EXP1-2391

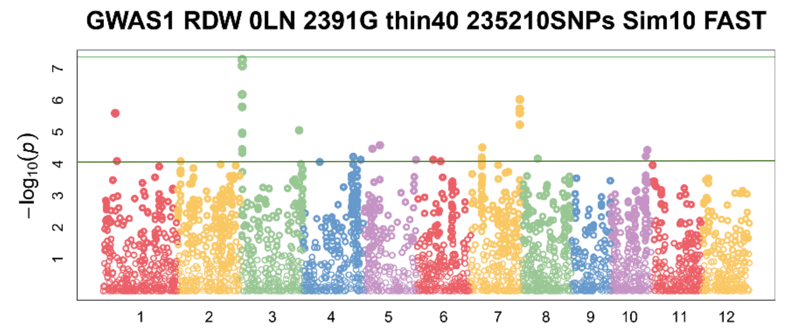

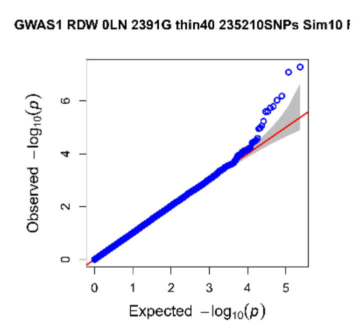

EXP1-1332

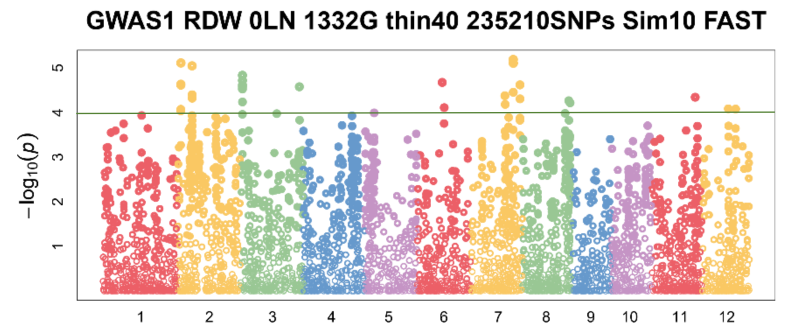

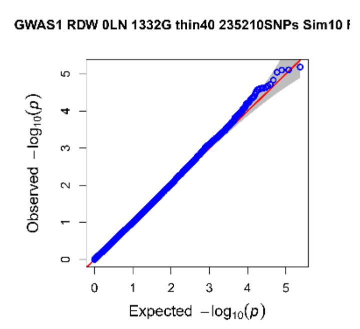

EXP2-1332

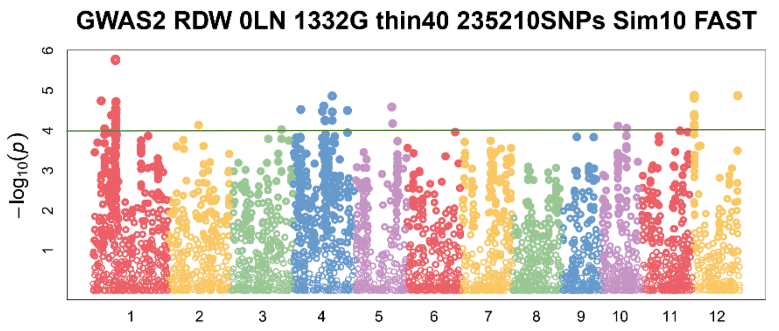

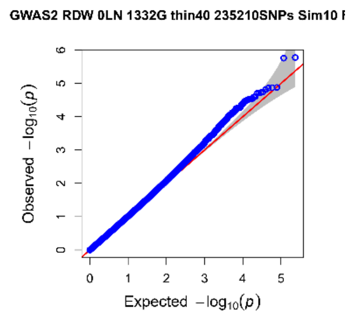

2.4. Ratio of shoot dry weight and root dry weight

EXP1-2391

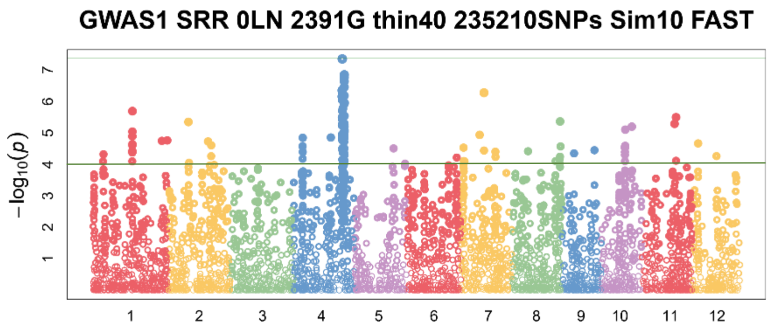

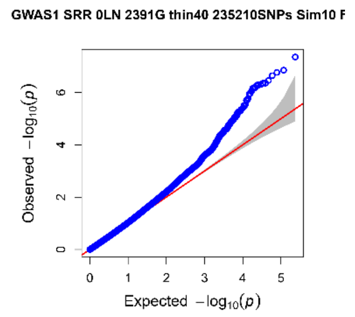

EXP1-1332

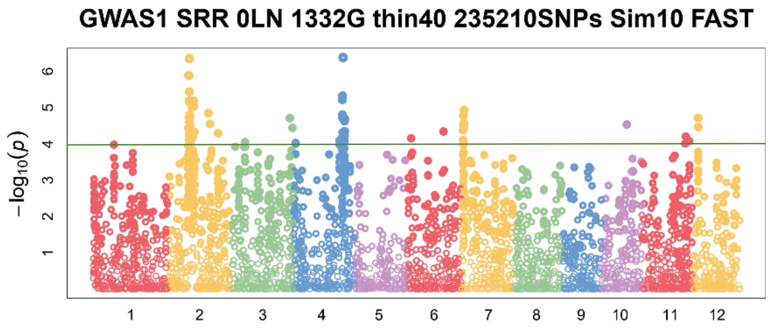

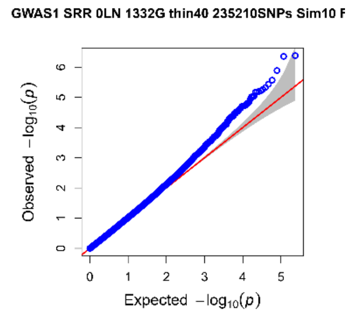

EXP2-1332

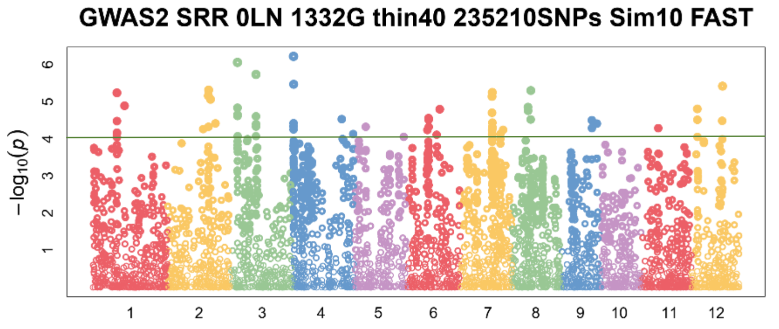

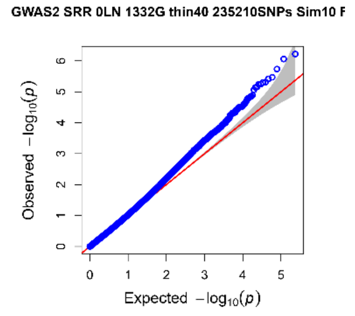

3. Treatment of 60 NaCl x standard N (SN-60Na)

3.1. Plant dry weight (PDW)

EXP1-2391	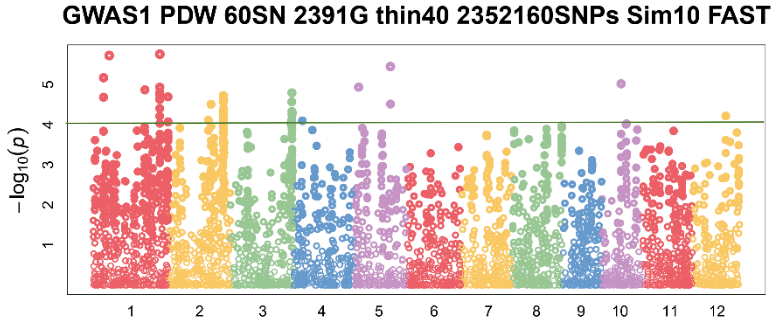	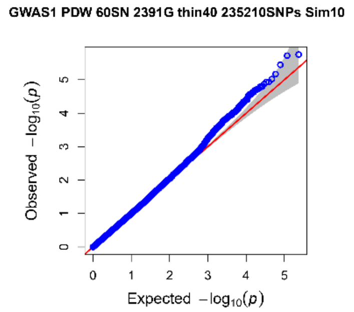
EXP1-1332	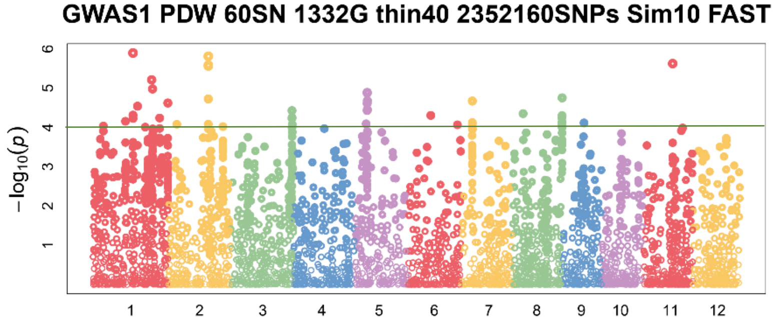	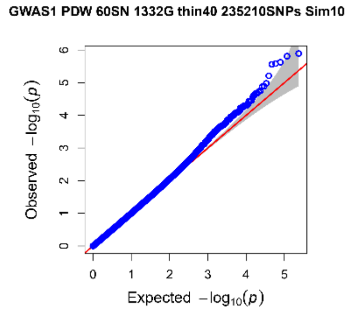
EXP2-1332	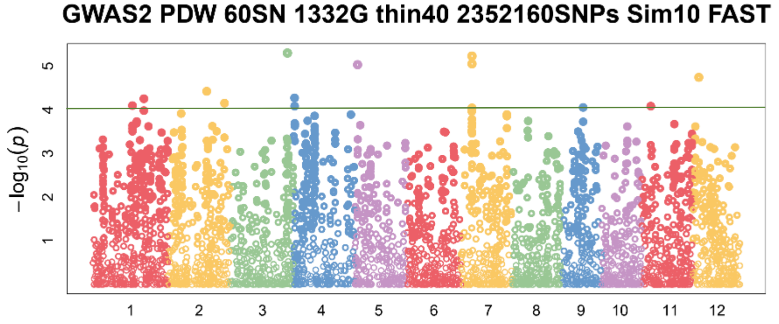	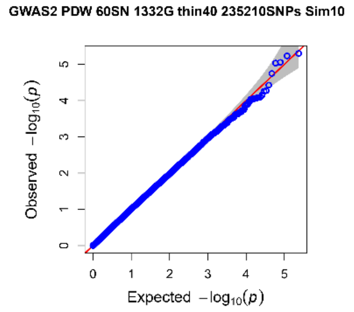

3.2. Shoot dry weight (SDW)

EXP1-2391	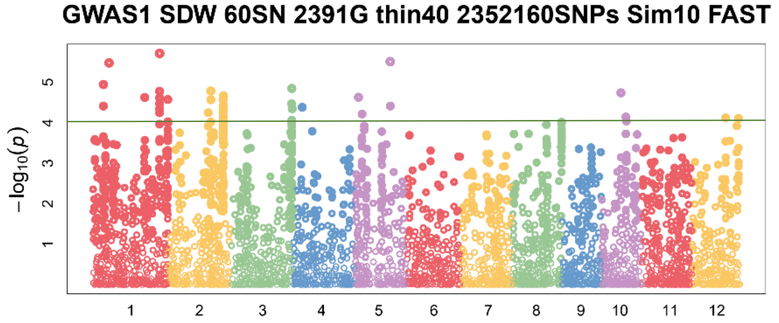	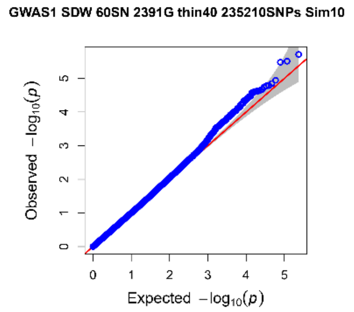

EXP1-1332

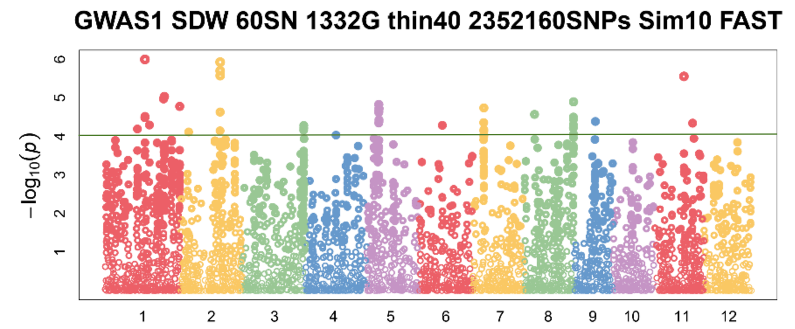

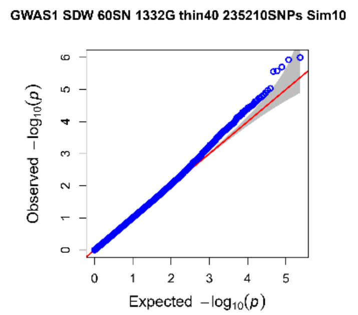

EXP2-1332

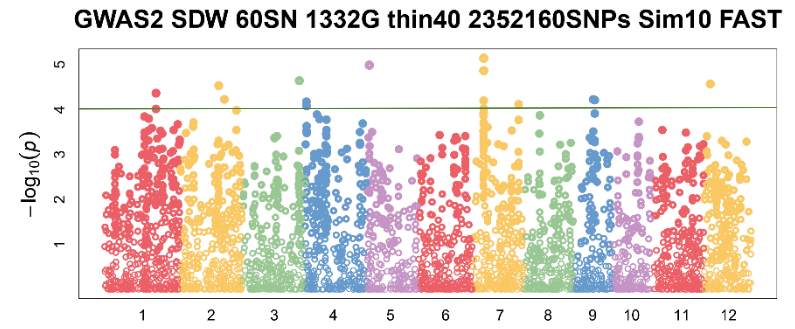

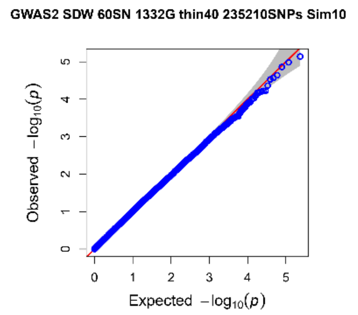

3.3. Root dry weight (RDW)

EXP1-2391

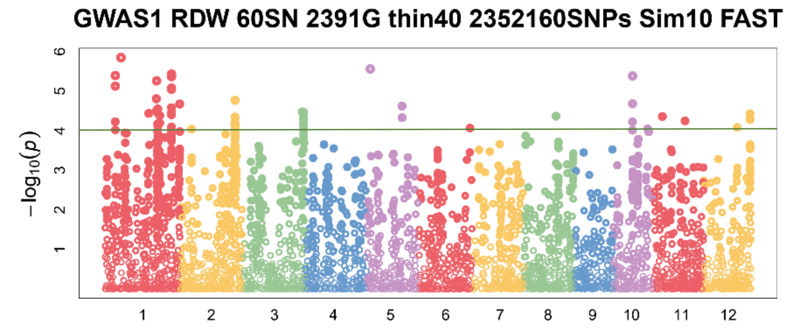

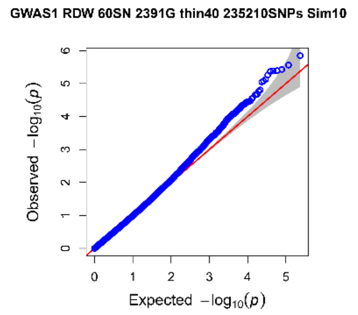

EXP1-1332

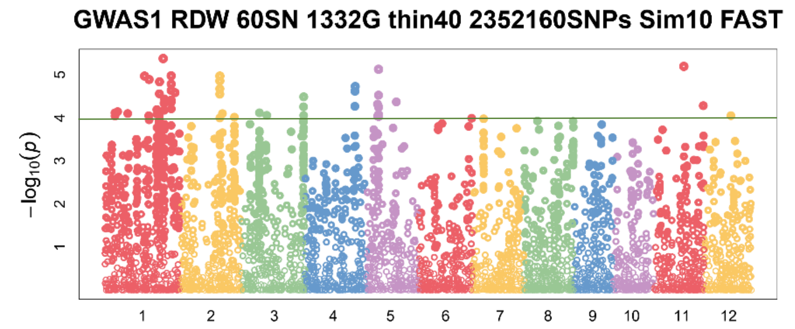

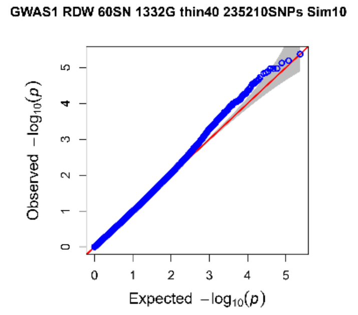

EXP2-1332	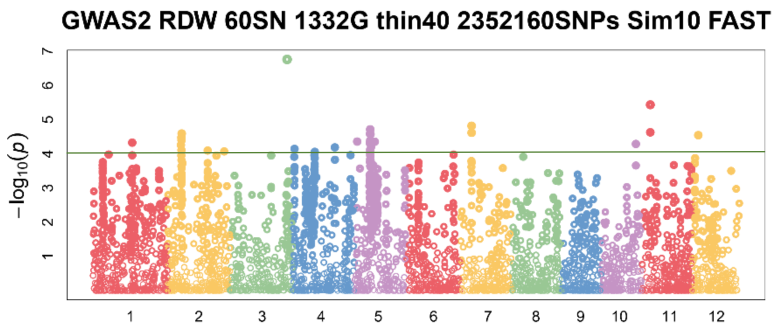	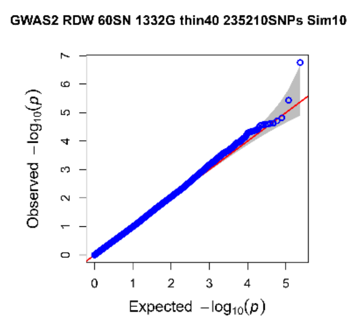

3.4. Ratio of shoot dry weight and root dry weight

EXP1-2391	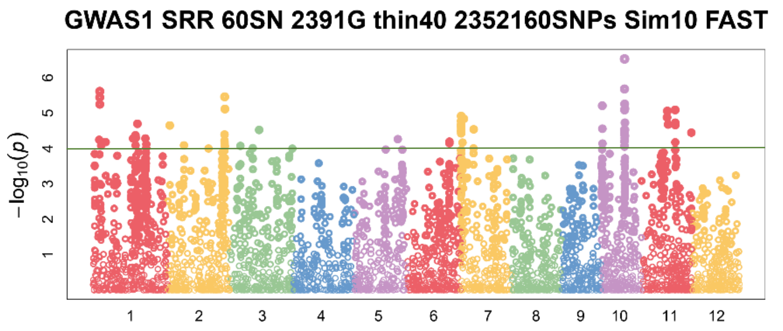	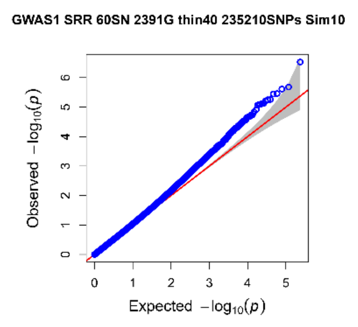
EXP1-1332	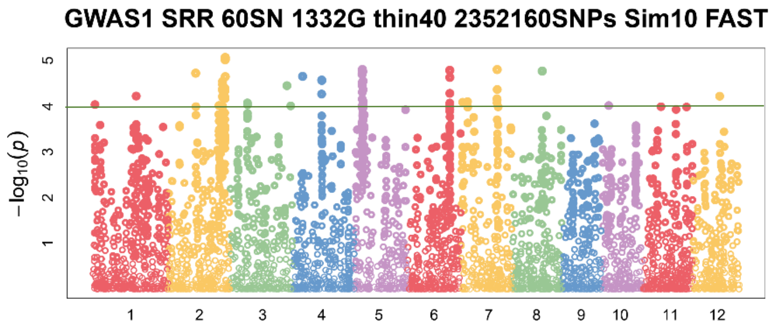	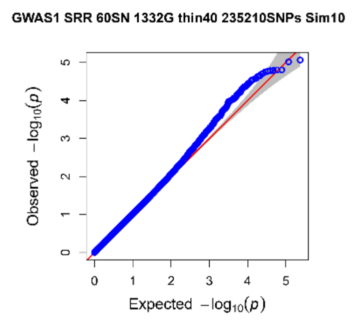
EXP2-1332	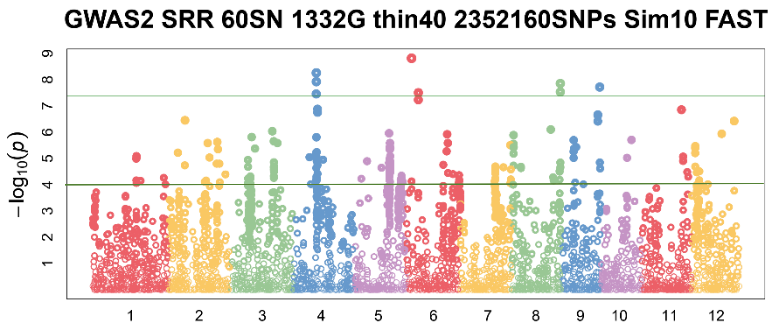	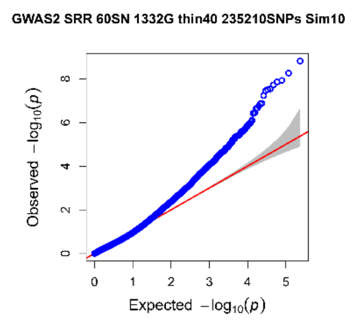

4. Treatment of 60 NaCl x low N (LN-0Na)

4.1. Plant dry weight (PDW)

EXP1-2391	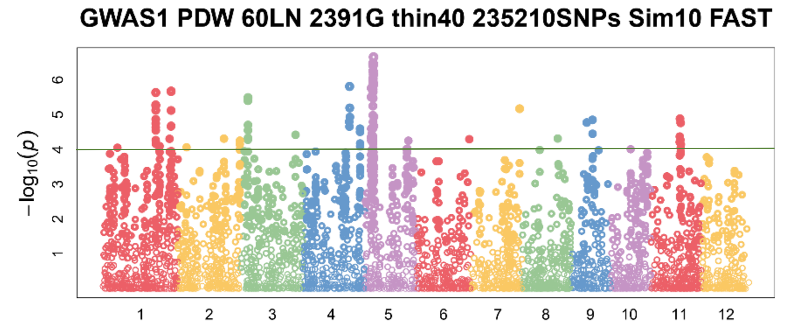	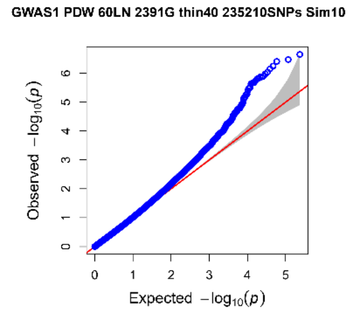
EXP1-1332	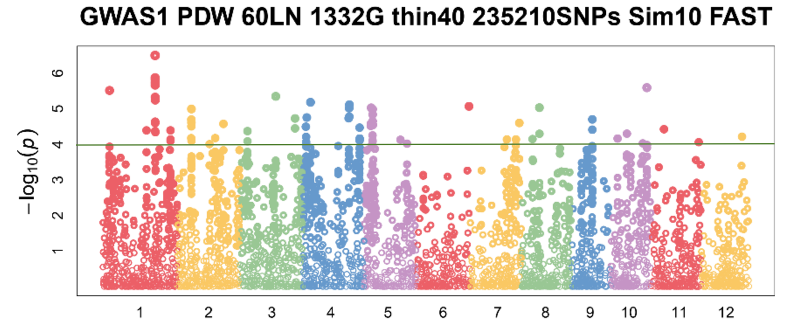	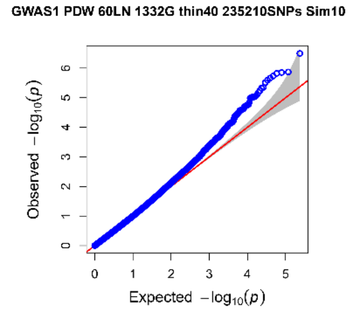
EXP2-1332	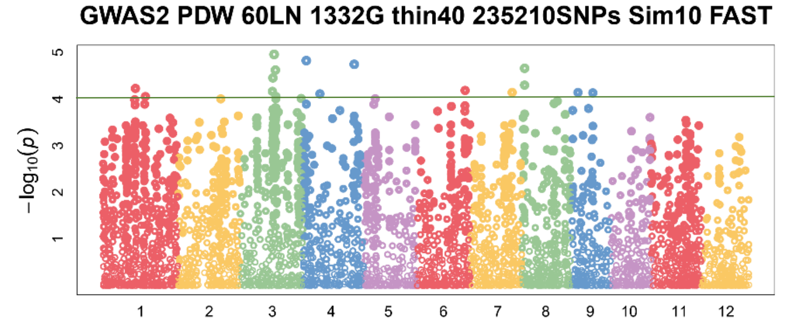	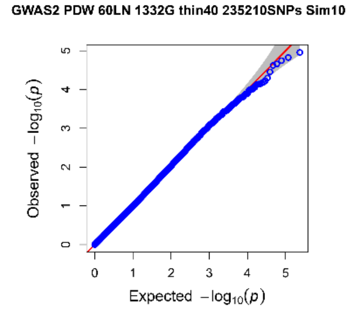

4.2. Shoot dry weight (SDW)

EXP1-2391	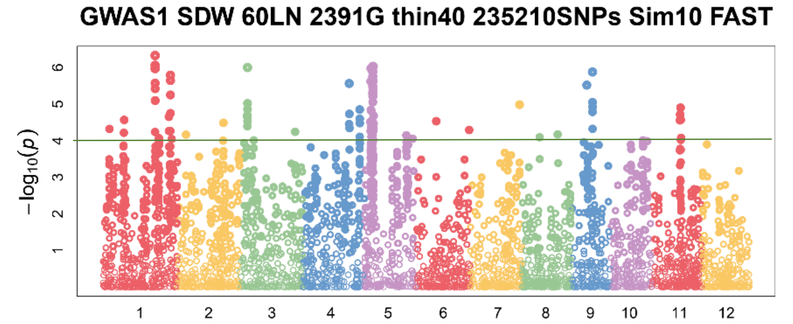	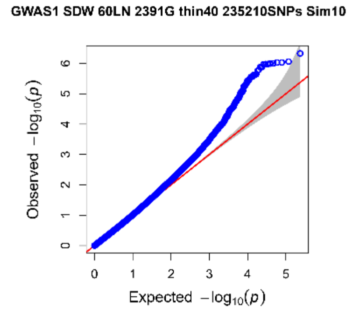

EXP1-1332	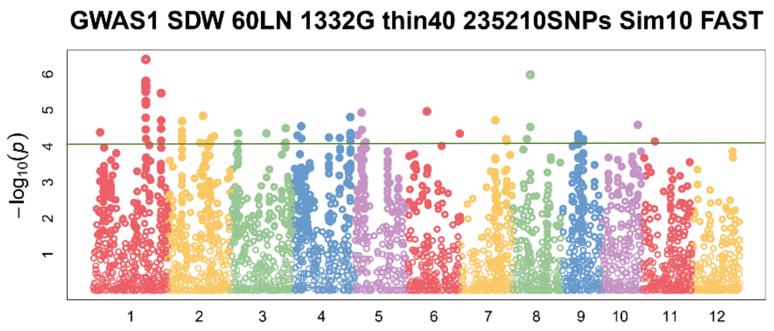	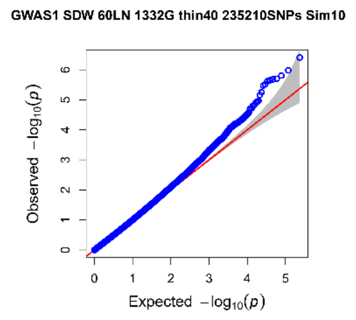
EXP2-1332	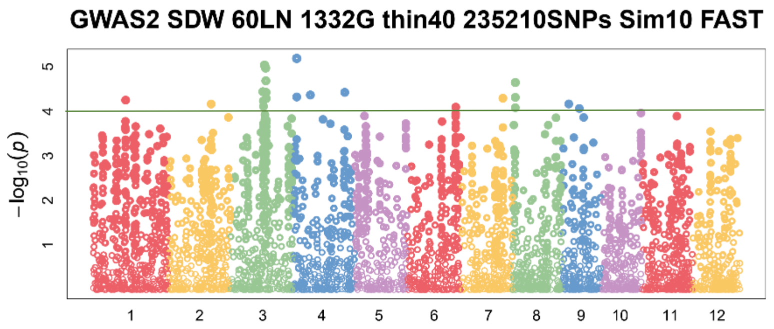	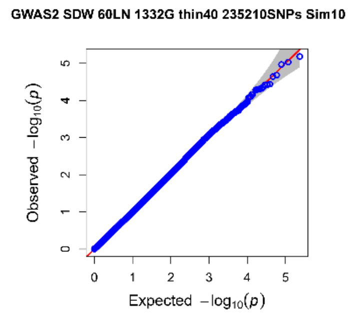

4.3. Root dry weight (RDW)

EXP1-2391	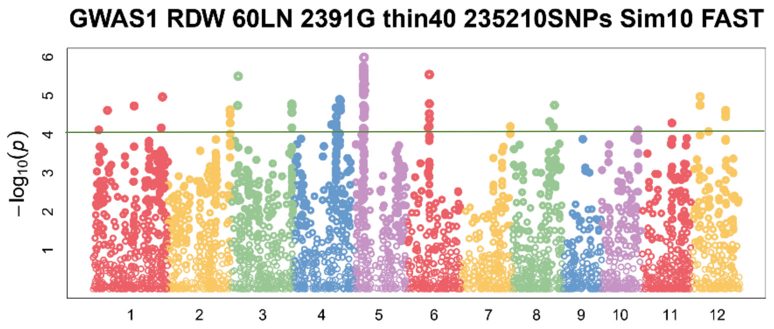	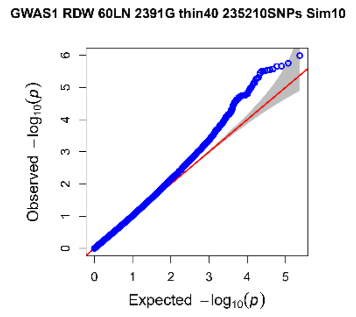
EXP1-1332	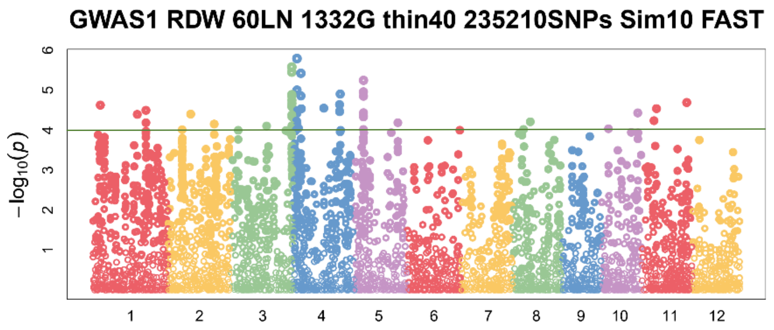	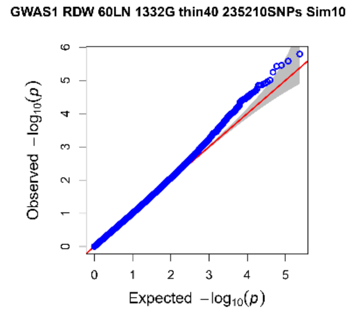

EXP2-1332	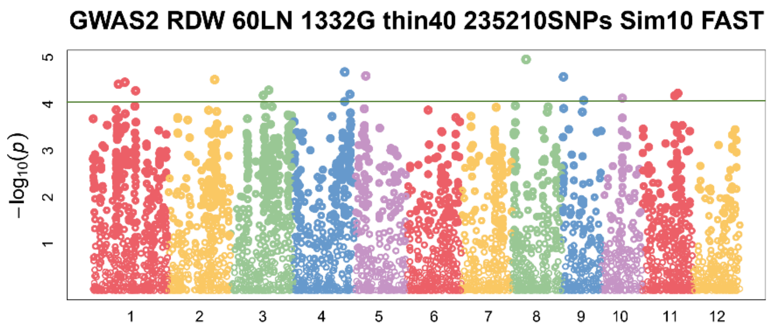	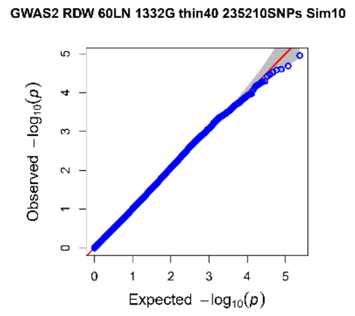

4.4. Ratio of shoot dry weight and root dry weight

EXP1-2391	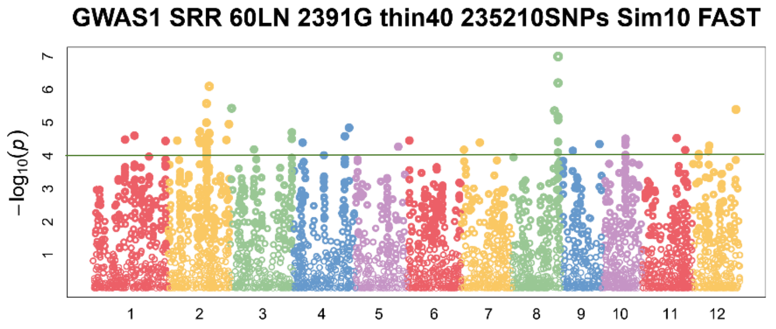	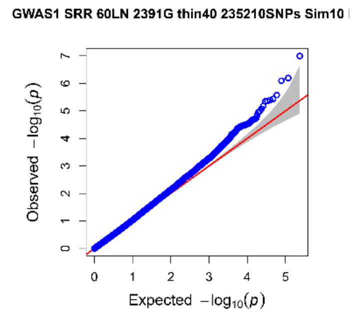
EXP1-1332	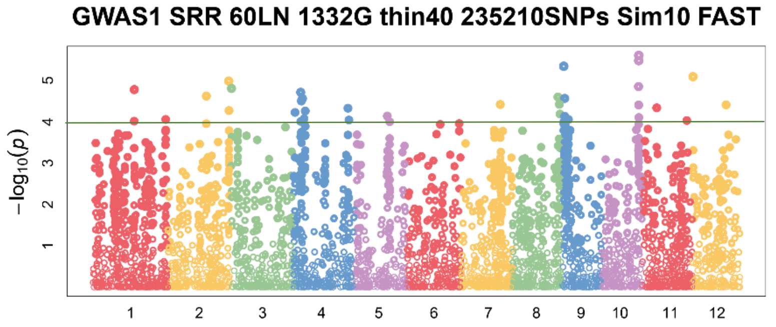	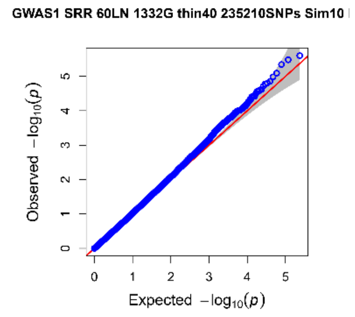
EXP2-1332	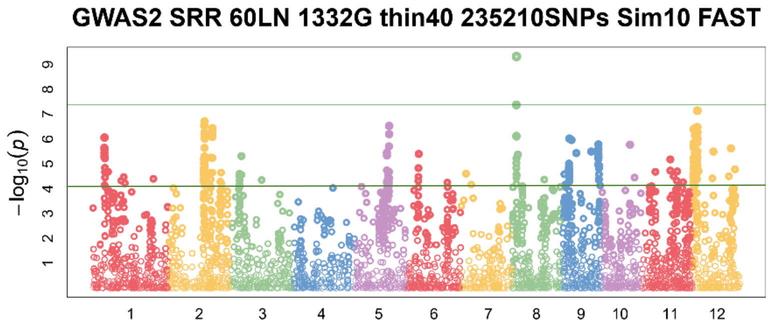	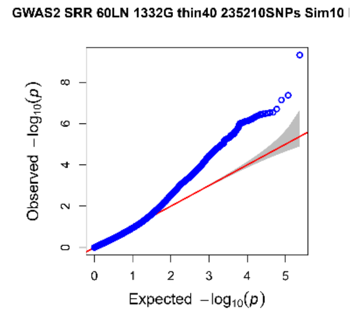

5. Relative $PDW_{LN\text{-}0Na/SN\text{-}0Na}$

EXP1-2391	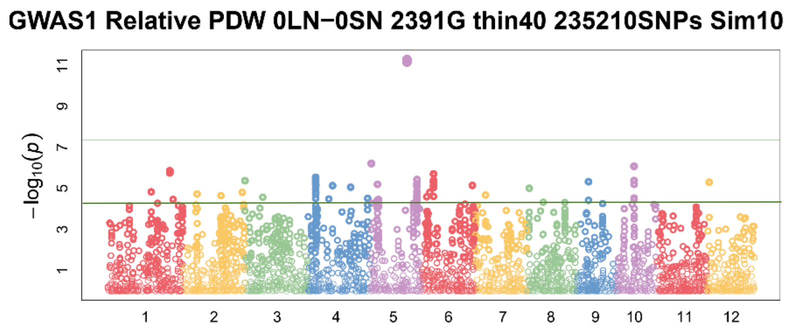	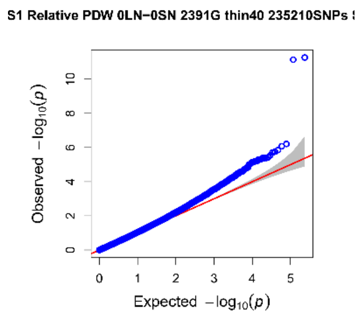
EXP1-1332	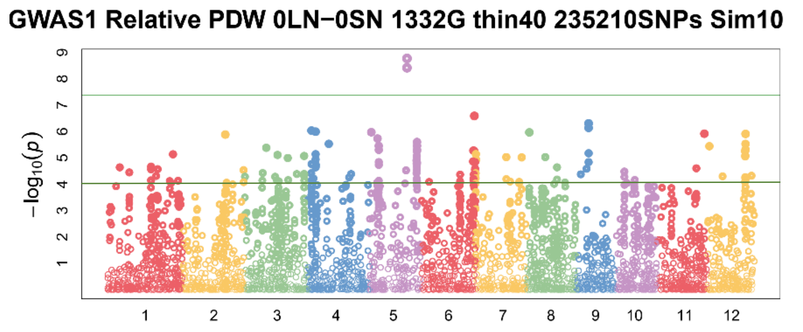	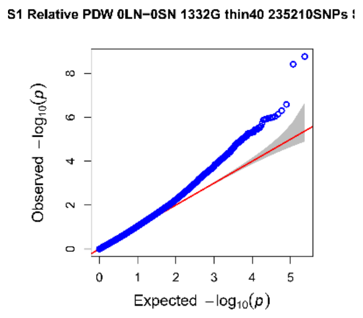
EXP2-1332	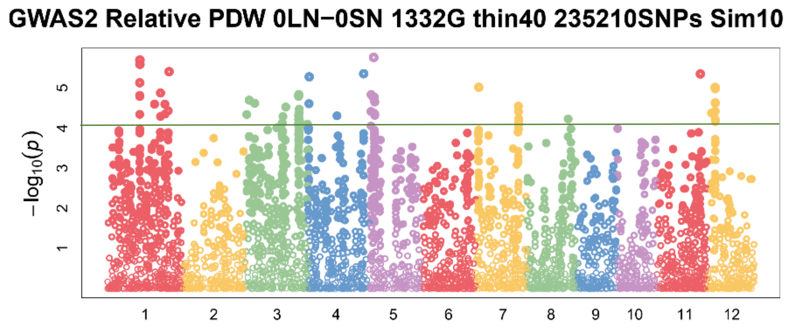	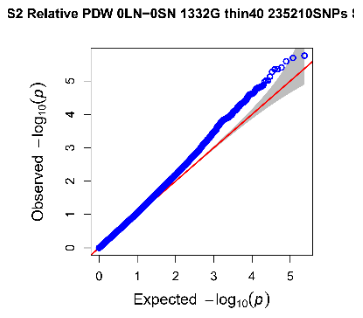

6. Relative $PDW_{SN\text{-}60Na/SN\text{-}0Na}$

EXP1-2391	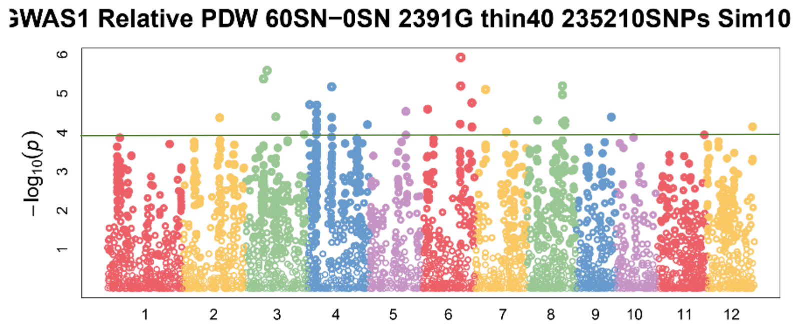	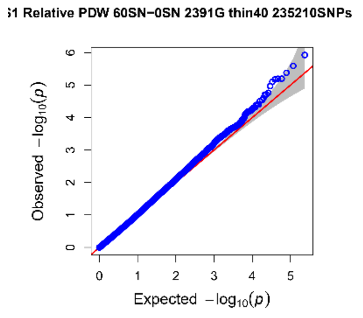

EXP1-1332	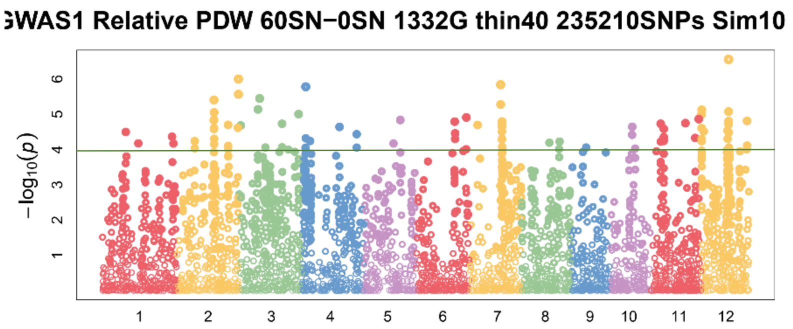	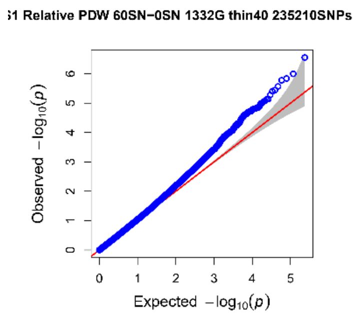
EXP2-1332	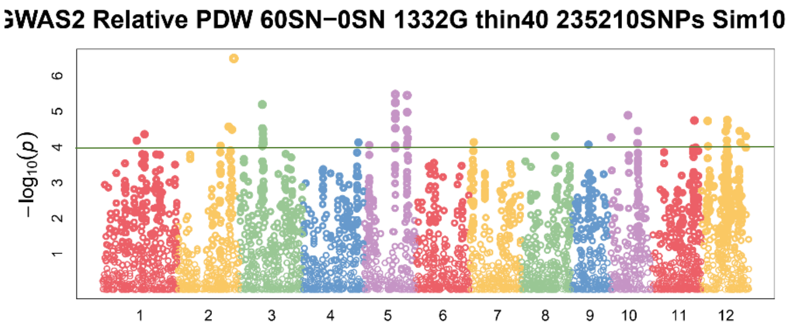	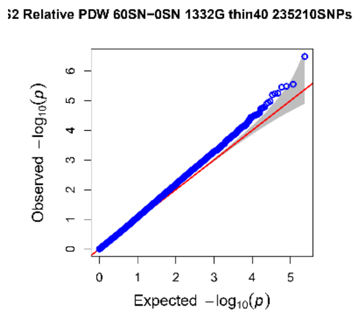

7. Relative $PDW_{LN-60Na/LN-0Na}$

EXP1-2391	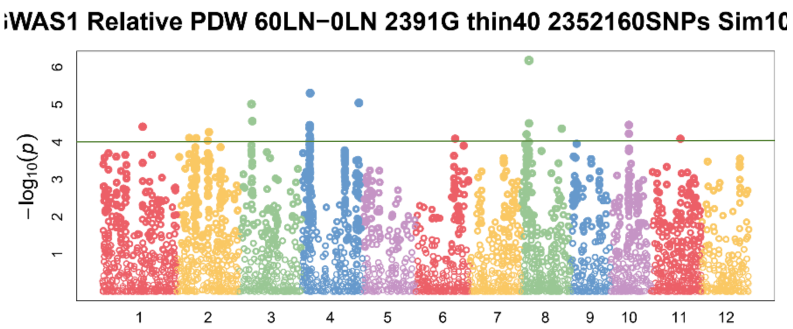	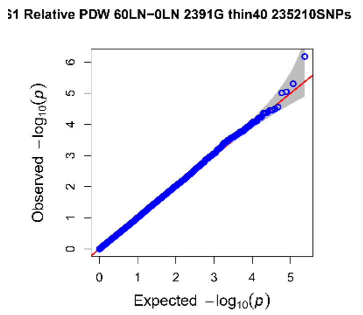
EXP1-1332	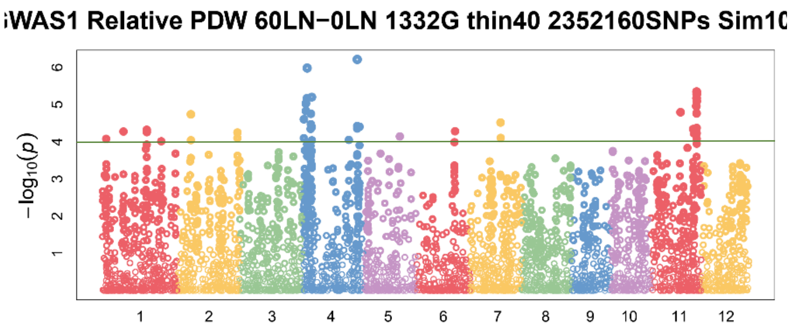	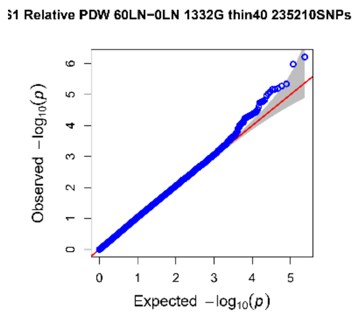

EXP2-1332

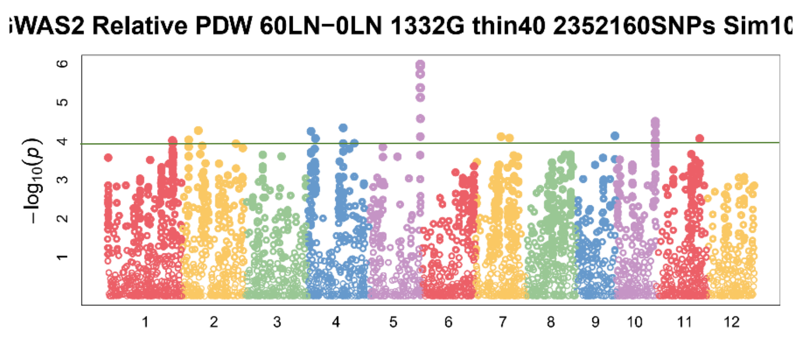

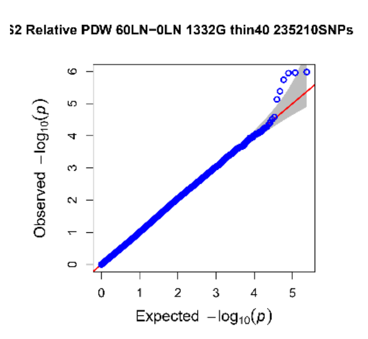

8. Relative $PDW_{LN-60Na/SN-60Na}$

EXP1-2391

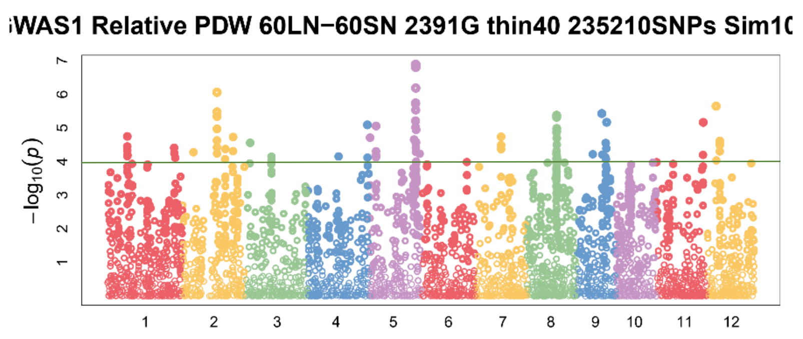

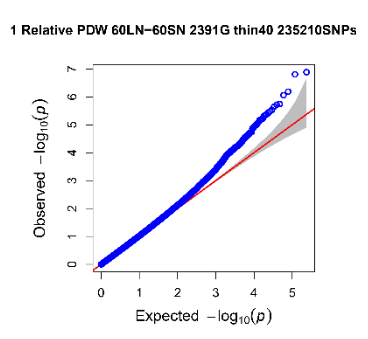

EXP1-1332

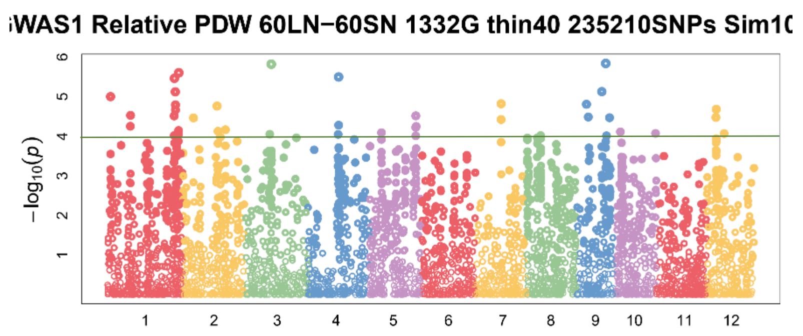

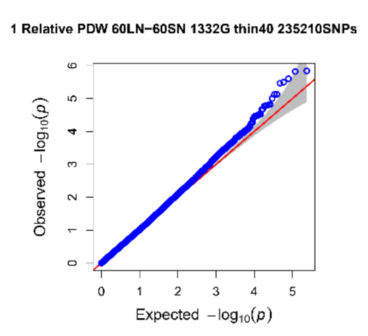

EXP2-1332

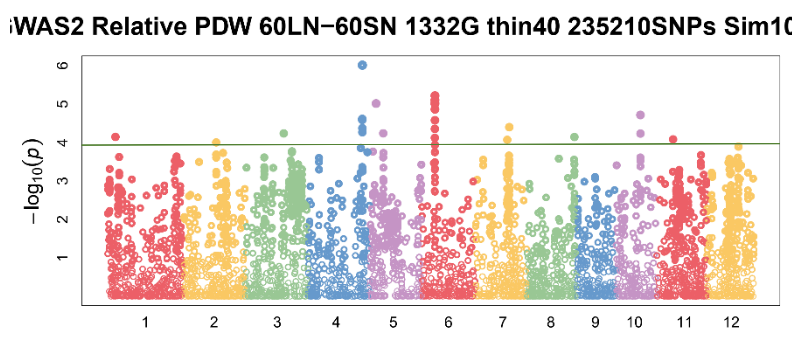

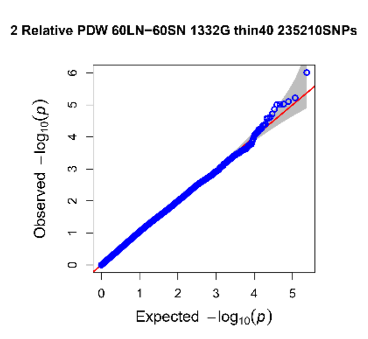

Fig. S4 Manhattan plot and Q-Q plot of rice accessions in different treatments in different runnings
